# C-Myc-activated long non-coding RNA LINC01050 promotes gastric cancer growth and metastasis by sponging miR-7161-3p to regulate SPZ1 expression

**DOI:** 10.1186/s13046-021-02155-7

**Published:** 2021-11-08

**Authors:** Ziwei Ji, Tianbin Tang, Mengxia Chen, Buyuan Dong, Wenjing Sun, Nan Wu, Hao Chen, Qian Feng, Xingyi Yang, Rong Jin, Lei Jiang

**Affiliations:** 1 Department of Gastroenterology, The First Affiliated Hospital of Wenzhou Medical University, Wenzhou, 325000 China; 2 Central Laboratory, The First Affiliated Hospital of Wenzhou Medical University, Wenzhou, 325000 China

**Keywords:** Gastric cancer, C-Myc, LINC01050, miR-7161-3p, SPZ1, Metastasis

## Abstract

**Background:**

Growing evidence shows that long non-coding RNAs (lncRNAs) play significant roles in cancer development. However, the functions of most lncRNAs in human gastric cancer are still not fully understood. Here, we explored the role of a novel c-Myc-activated lncRNA, LINC01050, in gastric cancer progression.

**Methods:**

The expression of LINC01050 in the context of gastric cancer was assessed using The Cancer Genome Atlas datasets. Its functions in gastric cancer were investigated through gain- and loss-of-function experiments combined with the Cell Counting Kit-8 assays, colony-forming assays, Transwell assays, flow cytometry, Western blot analyses, and xenograft tumor and mouse metastasis models. Potential LINC01050 transcription activators were screened via bioinformatics and validated by chromatin immunoprecipitation and luciferase assays. The interaction between LINC01050 and miR-7161-3p and the targets of miR-7161-3p were predicted by bioinformatics analysis and confirmed by a luciferase assay, RNA immunoprecipitation, RNA pull-down, and rescue experiments.

**Results:**

LINC01050 was significantly up-regulated in gastric cancer, and its high expression was positively correlated with a poor prognosis. The transcription factor c-Myc was found to directly bind to the LINC01050 promoter region and activate its transcription. Furthermore, overexpression of LINC01050 was confirmed to promote gastric cancer cell proliferation, migration, invasion, and epithelial-mesenchymal transition in vitro and tumor growth in vivo. At the same time, its knockdown inhibited gastric cancer cell proliferation, migration, invasion, and epithelial-mesenchymal transition in vitro along with tumor growth and metastasis in vivo*.* Moreover, mechanistic investigations revealed that LINC01050 functions as a molecular sponge to absorb cytosolic miR-7161-3p, which reduces the miR-7161-3p-mediated translational repression of SPZ1, thus contributing to gastric cancer progression.

**Conclusions:**

Taken together, our results identified a novel gastric cancer-associated lncRNA, LINC01050, which is activated by c-Myc. LINC01050 may be considered a potential therapeutic target for gastric cancer.

**Supplementary Information:**

The online version contains supplementary material available at 10.1186/s13046-021-02155-7.

## Background

Gastric cancer (GC) is the third leading cause of cancer-related death worldwide due to the combination of its high incidence and a lack of effective treatment options [1]. GC is often diagnosed in the middle- or late-stage and is accompanied by malignant proliferation and metastasis in most patients. Even with significant advances in surgical techniques, diagnosis and molecular targeting therapy, the prognosis of advanced-stage patients remains very poor [2–4]. As such, a better understanding of the molecular mechanism of GC progression is necessary to provide potential biomarkers and targets for improving the diagnosis and treatment of GC.

To date, most studies have mainly focused on protein-coding genes. However, human genome sequencing data reveals that protein-coding sequences occupy less than 2% of the human genome, and 98% are non-coding RNAs [5, 6]. Long non-coding RNAs (lncRNAs) are a class of non-coding RNAs with transcripts that are > 200 nt long and have limited or no protein-coding potential [7]. Despite not encoding proteins, lncRNAs have been revealed to play essential roles in tumorigenesis [7] and regulating the expression of potential target genes at the epigenetic, transcriptional, and post-transcriptional levels [8, 9]. LncRNAs also play key roles in critical biological processes, such as chromosome imprinting, stem cell differentiation, immune response, tumorigenesis, and chemotherapy resistance [10–12]. Recently, numerous lncRNAs were revealed to be associated with human diseases, especially cancer [13]. However, the role of lncRNAs in the development of GC is explicitly not well understood.

In this study, we first identified a novel GC-associated lncRNA, LINC01050, which is activated by c-Myc. We found that LINC01050 was significantly up-regulated in GC tissues compared with the corresponding non-tumor tissues, and its expression may serve as a potential independent predictor for overall survival in GC. Moreover, we determined that LINC01050 regulated GC progression and metastasis by functioning as a competing endogenous RNA (ceRNA) for miR-7161-3p, thereby preventing the latter’s association with its target *SPZ1*. Our data indicate that LINC01050 plays a critical role in GC progression and is a potential candidate for GC diagnosis and treatment.

## Materials and methods

### Data treating

The data of RNA expression profiles for stomach adenocarcinoma (STAD) were downloaded from Xena platform [14], including 375 STAD tissues and 32 non-tumor tissues. The lncRNAs were annotated by the human gene annotation files (GRCh38.90), which was downloaded from the Ensembl database (https://asia.ensembl.org). The lncRNAs were defined as 3-prime overlapping ncRNAs, anti-sense RNAs, bidirectional promoter lncRNAs, long intergenic non-coding RNAs (lincRNAs), macro lncRNAs, non-coding, processed transcript, sense intronic, and sense overlapping. The lncRNA expression data were analyzed by the R/Bioconductor package DESeq2 [15]. The clinical data information for STAD were collected using R/Bioconductor package TCGAbiolinks [16].

### Human tissue samples

Tissue from 29 GC cases was obtained with the written consent of patients who underwent surgery at the First Affiliated Hospital of Wenzhou Medical University. The Ethics Committee of the First Affiliated Hospital of Wenzhou Medical University approved this study.

### Cell culture

The human GC cell lines (AGS, BGC-823, and KATO III) and the HEK293T cell line were purchased from the Typical Culture Collection of the Chinese Academy of Sciences (Shanghai, China). The AGS, BGC823, and KATO III cells were cultured in RPMI 1640 (Life Technologies, Carlsbad, CA, USA) supplemented with 10% fetal bovine serum (FBS) (Sigma-Aldrich, St Louis, MO, USA). The HEK293T cells were cultured in Dulbecco’s Modified Eagle’s Medium (DMEM) (Life Technologies) supplemented with 10% FBS. The cells were cultured in a humidified 37 °C incubator supplemented with 5% CO_2_.

### Cell transfection

Transfection was performed using Lipofectamine 3000 (Life Technologies) according to the manufacturer’s protocol. The miR-7161-3p mimic, LINC01050 siRNA, c-Myc siRNA, SPZ1 siRNA, and scramble siRNA (si-NC) were purchased from Guangzhou Ruibo Biotechnology Co., Ltd. (Guangzhou, China). The nucleotide sequences of the siRNAs are listed in Additional file 1: Table S1.

### Lentiviral vector construction and transduction

The human LINC01050 transcript cDNA was amplified in the BGC-823 cells and was cloned into the lentiviral vector pLVX-puro by digesting it with EcoRI and BamHI. A short hairpin RNA directed against LINC01050 (sh-LINC01050) was inserted into the pLKO.1 puro vector that was digested with AgeI and EcoRI. The lentiviruses were generated by the transient transfection of the transfer vector and three packaging vectors (pMDLg/pRRE, pRSV-REV, and pCMV-VSVG) into HEK293T cells. The GC cells were transduced with lentiviruses expressing LINC01050, sh-LINC01050, or the negative control.

### Quantitative reverse transcription-polymerase chain reaction (qRT-PCR)

The total RNA was extracted from the cells using the TRIzol Reagent (Thermo Fisher Scientific, Waltham, MA, USA), and 1 μg of total RNA was used for cDNA synthesis using the RevertAid First Strand cDNA Synthesis Kit (Thermo Fisher Scientific) according to the manufacturer’s protocol. The expression levels of LINC01050, miR-7161-3p, SPZ1, and c-Myc were evaluated by qRT-PCR using SYBR Premix ExTaq (Takara, Japan) and the QuantStudio 5 real-time PCR system (Applied Biosystems, Warrington, UK). After an initial activation at 95 °C for 30 s, 40 PCR cycles were performed using the following conditions: denaturation at 95 °C for 5 s and annealing/extension at 60 °C for 34 s. The U6 gene was used to normalize the expression level of miR-7161-3p. GAPDH was used to normalize the expression levels of LINC01050, c-Myc, and SPZ1. The specific PCR primers and RT primers are presented in Additional file 1: Table S2.

### Isolation of cytoplasmic and nuclear RNA

Cytoplasmic and nuclear RNA was isolated using a PARIS Kit (Thermo Fisher Scientific, Waltham, MA, USA) according to the manufacturer’s protocol. The expression level of LINC01050 in the cytoplasm and nucleus was detected by qRT-PCR.

### Cell proliferation assays

Cell proliferation was assessed using the Cell Counting Kit-8 (CCK8) and ethynyl deoxyuridine (EdU) incorporation assays. After transfecting the cells with the siRNAs or miRNA mimics for 24 h, the GC cells were trypsinized and seeded into 96-well plates in a volume of 100 μl of complete medium (3000 cells/well). At 0, 24, 48, and 72 h after plating, 10 μl of the CCK8 solution (Dojindo, Japan) was added to each well. After an 4 h incubation, each well was measured at 450 nm according to the manufacturer’s instructions. EdU cell proliferation staining was performed using the BeyoClick™ EdU Cell Proliferation Kit with Alexa Fluor 488 (Beyotime, China). Briefly, the cells were incubated with EdU for 2 h, fixed with 4% paraformaldehyde and permeated with 0.3% Triton X-100. Then the cells were incubated with the Click Reaction Mixture for 30 min at room temperature in the dark and stained with Hoechst. The stained cells were photographed by fluorescence microscopy (Leica, Wetzlar, Germany).

The GC cells transfected with si-LINC01050 were plated in 6-well plates at a density of 2000 cells/well for the plate colony formation assay. After 2 weeks, the colonies were fixed for 30 min with 4% paraformaldehyde and were stained for 15 min with 0.1% crystal violet. The plate colony formation was determined by counting the number of colonies. All the experiments were repeated three times.

### Western blot analysis

The GC cells were lysed in RIPA buffer (Thermo Fisher Scientific) supplemented with protease and phosphatase inhibitors (Thermo Fisher Scientific). The total protein concentration was measured by the Pierce BCA Protein Assay Kit (Thermo Fisher Scientific) according to the manufacturer’s protocol. The total cellular protein was separated by 12% sodium dodecyl sulfate-polyacrylamide gel electrophoresis. Subsequently, the electrophoresed proteins were transferred to a polyvinylidene fluoride membrane and were blocked with 5% skimmed milk for 2 h at room temperature. Then, the membrane was incubated with the primary antibodies overnight at 4 °C and the secondary antibody for 1 h at room temperature. The specific bands were detected using an enhanced chemiluminescence detection system (Bio-Rad, California, CA, USA). The following primary antibodies were used: mouse anti-vimentin (diluted 1:5000; Cat. #550513; BD Biosciences, San Jose, CA, USA); rabbit anti-E-cadherin (diluted 1:1000; Cat. #3195S; Cell Signaling Technology, Danvers, MA, USA); rabbit anti-Cleaved PARP1 (diluted 1:1000; Cat. # ab32064; abcam); rabbit anti-Cleaved Caspase-3 (diluted 1:1000; Cat. #9664; Cell Signaling Technology); mouse anti-Bcl-2 (diluted 1:1000; Cat. #15071; Cell Signaling Technology); mouse anti-Bax (diluted 1:1000; Cat. #89477; Cell Signaling Technology); rabbit anti-GAPDH (diluted 1:1000; Cat. #5174S; Cell Signaling Technology); rabbit anti-SPZ1 (diluted 1:1000; Cat. #DF8886; Affinity Biosciences, OH, USA); rabbit anti-AGO2 (diluted 1:1000; Cat. # ab186733; abcam); and rabbit anti-c-Myc (diluted 1:1000; Cat. #18583S; Cell Signaling Technology).

### Cell migration and invasion assays

The cell migration and invasion assays were performed using a Transwell chamber (Costar; Corning Incorporated, Cambridge, MA, USA) according to the manufacturer’s instructions. For the migration assay, the cells (1.5 × 10^5^/200 μL) were seeded onto the Transwell filter membrane chambers in cell culture medium without FBS. Medium supplemented with 20% FBS was added to the lower chambers as a chemoattractant. After an 36 h incubation, the cells on the upper membrane were removed. The bottom surface was fixed with 4% paraformaldehyde for 20 min and was stained with a 0.1% crystal violet solution for 15 min. The number of cells that migrated to the lower chamber was counted (the fields were randomly selected under a light microscope at a magnification of × 10). For the invasion assay, the upper membranes were precoated with 10 μL of Matrigel (4.53 mg/mL; BD Biosciences, San Jose, CA, USA) before the process described above was carried out.

### Apoptosis assays

The KATO III and BGC823 cells were transfected with si-LINC01050 or si-NC for 48 h. Cell apoptosis was measured using an Annexin V-FITC/ Propidium iodide (PI) apoptosis detection kit (Multi Sciences, Hangzhou, China) according to the manufacturer’s protocol. After double staining with Annexin V-FITC (5 μL) and PI (10 μL), the cells were analyzed using a FACSCalibur flow cytometer (Becton Dickinson, Franklin Lakes, NJ, USA).

### Luciferase assay

To generate the LINC01050 promoter construct, the fragment (between − 740 and − 2000 bp) was amplified from HEK293T DNA and inserted into the pGL3-basic luciferase reporter vector (Promega, Madison, WI, USA). To construct the LINC01050 promoter mutation vector, the c-Myc binding site sequence was deleted in the corresponding LINC01050 promoter construct using the QuikChange Lightning Site-Directed Mutagenesis Kit (STRATAGENE, USA). The deletion was confirmed by sequencing. The pIRES2-c-Myc and pIRES2-vector were individually co-transfected into HEK293T cells together with the pGL3-based construct containing the LINC01050 WT or c-Myc deletion promoter sequences plus the Renilla plasmid (RL-SV40).

The complementary DNA fragment containing the wild type or mutant LINC01050 fragment and the 3’untranslated region (UTR) of SPZ1 was subcloned downstream of the luciferase gene within the pmirGLO luciferase reporter vector. HEK293T cells were co-transfected with the LINC01050-WT, LINC01050-MUT, SPZ1-WT, or SPZ1 MUT reporter plasmids individually and together with the miR-7161-3p mimics or NC mimics. At 48 h post-transfection, the firefly and Renilla luciferase activities were measured using a Dual-Luciferase Reporter Assay System (Promega). The ratio of the firefly luciferase to Renilla activity was calculated for each sample.

### Tumor growth and lung metastasis in nude mice

The Animal Experimental Ethics Committee of Wenzhou Medical University approved all the animal experiments. Four-to-six-week-old male athymic nude mice were purchased from the Zhejiang Charles River Laboratory Animal Co.Ltd. (Zhejiang, China). The nude mice were randomly grouped (*n* = 5 per treatment group) and were injected subcutaneously with 5 × 10^6^ KATO III cells transduced with lentiviral shNC or shLINC01050. The tumor length and width were measured using a vernier caliper every 5 days. The tumor volume (mm^3^) was calculated as follows: 0.5 × length × (width).^2^ The mice were euthanized, and the tumors were isolated on day 25. For the tumor metastasis experiment, 5 × 10^6^ BGC823 cells transduced with lentiviral shNC or shLINC01050 were suspended in 200 μL PBS and were injected into the tail vein of the athymic nude mice (*n* = 5 per group). The body weight of the mice was measured every 3 days. Forty-three days later, the mice were euthanized, and the lung metastases were evaluated.

### Immunohistochemistry

The streptavidin-biotin peroxidase complex method was used for immunohistochemical staining of the formalin-fixed, paraffin-embedded tissue sections. The tissue samples were dehydrated, embedded with paraffin, and cut into 4-μm-thick sections. The paraffin sections were dewaxed by dimethylbenzene and rehydrated by a gradually reduced concentration of ethanol. Antigen retrieval was performed by heating the dewaxed and dehydrated sections in an antigen retrieval solution containing 10 mM EDTA (pH 8.0) using a pressure cooker. Endogenous HRP activity was blocked with 3% H_2_O_2_. The primary antibodies were goat anti-human Ki-67 (ab16667, Abcam, USA; 1: 250 dilution) and mouse anti-human PCNA (ab29, Abcam, USA; 1: 10000 dilution). The sections were observed and photographed with an optical microscope (Leica, Wetzlar, Germany).

### Northern blot

LINC01050 northern blot was performed using a Roche DIG Northern Starter Kit (Roche, Switzerland) according to the manufacturer’s instructions. A total of 15 μg of RNA from each sample was subjected to formaldehyde gel electrophoresis and transferred to a HyBond N+ Nylon membrane (Amersham). The PCR primers used to generate the northern blot probe were 5′-GGAAGCAGCAAGGTCAATAC-3′ (forward) and 5′-AACAGGCT CCTCAAACAACT-3′ (reverse).

### RNA-fluorescence in situ hybridization (RNA-FISH)

RNA-FISH was performed to determine the subcellular location of LINC01050. The LINC01050 anti-sense FISH probe Mix was designed and synthesized by RiboBio (RiboBio Biotechnology, Guangzhou, China). According to the manufacturer’s protocol, the in situ hybridization was carried out with a fluorescent in situ hybridization (FISH) Kit. The fluorescence signals were scanned using the confocal laser microscope system (Leica, Wetzlar, Germany).

### Chromatin immunoprecipitation (ChIP) assays

The ChIP assay was performed using a ChIP assay kit (Millipore, Billerica, MA) according to the manufacturer’s protocol. Briefly, the KATO III cells were cross-linked with 1% formaldehyde for 10 min at 37 °C and were sonicated to shear the DNA to lengths between 200 and 1000 bp. Then, 10 μL of the supernatant was used as the input, and the remaining was diluted in the ChIP dilution buffer with protease inhibitor. The chromatin solution was incubated at 4 °C overnight with protein A + G magnetic beads coated with the anti-c-Myc antibody (3 μg) or IgG. A magnetic beads/antibody/histone complex was washed using a complex wash buffer, and the bead-bound immunocomplexes were eluted using an elution buffer. To reverse the histone-DNA crosslinks, the immune complexes were combined with 20 μL of 5 M NaCl, heated for 4 h at 65 °C, treated with proteinase K, and incubated at 45 °C for 1 h. The bound DNA fragments were purified and subjected to PCR using the specific primers. The specific PCR primers are listed in Additional file 1: Table S3.

### RNA immunoprecipitation (RIP)

RIP was performed using the EZ-Magna RIP™ RNA-Binding Protein Immunoprecipitation Kit (Millipore, Billerica, MA, USA) according to the manufacturer’s protocol. An AGO2 antibody (Abcam, ab32381) and the corresponding IgG were used for the immunoprecipitation. The co-precipitated RNAs were detected by real-time PCR.

### RNA pull-down assay

The LINC01050 biotin-labeled RNA probes were transcribed with a biotin RNA labeling mix (Roche, Switzerland) and T7 RNA polymerase (Roche, Switzerland) and treated with RNase-free DNase I (Promega, Madison, WI, USA) in vitro. After purification, the biotinylated RNAs were incubated with the cell lysate at 37 °C for 1 h. M-280 Streptavidin magnetic beads (Invitrogen, USA) were added to the KATO III cell lysate, and the mix was incubated at room temperature for 30 min with rotation. A Western blot assay was used to determine the AGO2 protein expression.

For the RNA-RNA pull-down assay, the cell lysate was incubated with the biotin-labeled LINC01050 using a Biotin RNA Labeling System at 4 °C overnight. The M-280 beads were added later. The co-immunoprecipitated RNAs were washed with buffers and purified. The purified miR-7161-3p RNAs were analyzed by qRT-PCR.

### Statistical analysis

All the experimental data are expressed as the mean ± standard deviation (SD). The statistical analyses were performed using SPSS 21.0 software (SPSS Inc., Chicago, IL, USA) or GraphPad Prism 5 (GraphPad Software Inc., La Jolla, CA, USA). Statistically significant differences were calculated using an independent sample *t*-test. *P* < 0.05 indicated a statistically significant difference.

## Results

### LINC01050 is up-regulated in human GC tissues and correlates with poor prognosis

**Fig. 1:**
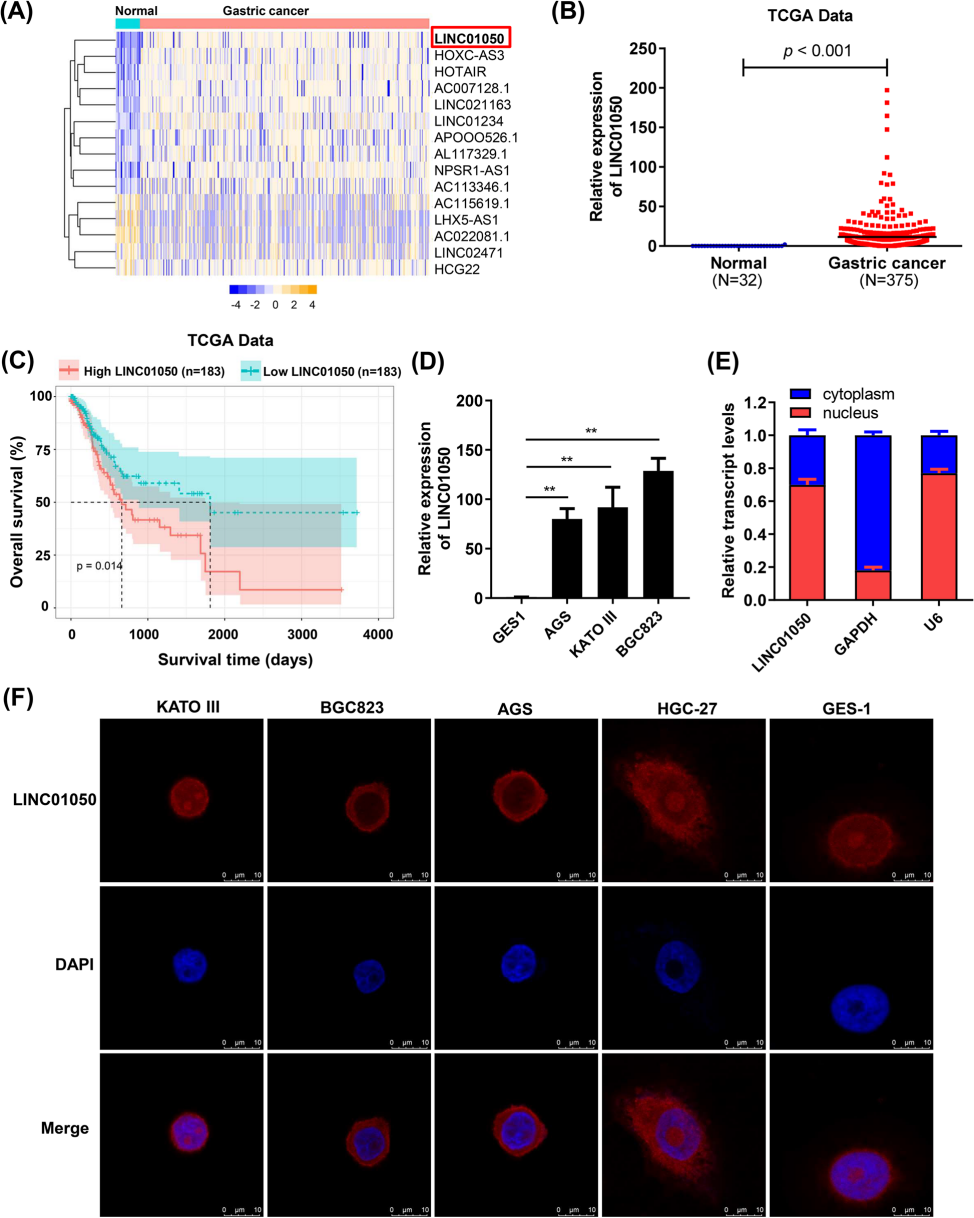
LINC01050 expression is up-regulated in gastric cancer tissues and is associated with a poor prognosis. **a** The top 15 dysregulated lncRNAs in gastric cancer from TCGA data. **b** Relative expression of LINC01050 in GC tissues compared with noncancerous tissues, based on TCGA data. *P* < 0.001. **c** Patients with high expression of LINC01050 showed reduced survival times compared to a low expression of LINC01050 (*p* = 0.014). **d** qRT-PCR analysis of LINC01050 expression in a normal gastric epithelium cell line (GES-1) and gastric cancer cell lines (AGS, KATO III, and BGC823). Data are presented as mean ± SD (*n* = 3). ^**^P < 0.01. **e** qRT-PCR analysis of LINC01050 expression in the nuclear and cytoplasmic fractions from KATO III cells. GAPDH was used as the cytoplasmic control, and U6 as the nucleus control. The data are represented as the mean ± SD (*n* = 3). **f** RNA-FISH detection of LINC01050 (red) in GC cell lines (KATO III, BGC823, AGS, and HGC-27) and GES-1 cells. The nuclei were counterstained using DAPI (blue). FISH, fluorescence in situ hybridization. Scale bar = 10 μm

To identify GC-related lncRNAs that may be associated with gastric tumorigenesis, we analyzed RNA sequencing data from 375 GC tissues and 32 adjacent non-tumor tissues in TCGA datasets (TCGA-stomach adenocarcinoma STAD). We identified 1022 lncRNAs that were differently expressed between GC and normal tissue, of which the top 15 are depicted in Fig. 1a. LINC01050 was up-regulated in the GC tissue (Fig. 1b). A Kaplan-Meier survival analysis revealed that patients with higher LINC01050 levels had shorter overall survival times than those with lower levels (Fig. 1c). We also measured the expression of LINC01050 by qRT-PCR in GC cell lines (AGS, KATO III, and BGC823) and the normal gastric epithelial cell line GES-1. The results showed that LINC01050 expression was significantly up-regulated in GC cells compared with the normal cell line (Fig. 1d). The subcellular fractionation and real-time PCR analysis showed that LINC01050 was localized at the cytoplasm and nucleus (Fig. 1e). In addition, the subcellular localization of LINC01050 was confirmed by RNA-FISH in GC cell lines (KATO III, BGC823, AGS, HGC-27) and the GES-1 cells (Fig. 1f). Finally, we predicted the coding ability of LINC01050 using the following five bioinformatic tools: CPAT [17]; CPC2 [18, 19]; RNAsamba [20]; LGC web server [21]; and CNIT [22], as previously described [23]. None displayed a positive result. More specifically, the first three tools showed coding probabilities of 0.01, 0.34, and 0.11, respectively, where a probability close to 1 indicates full coding potential. The latter two tools gave coding scores of − 0.43 and − 0.38, respectively, where a score larger than 0 represents a coding lncRNA. These analyses suggested that LINC01050 does not have protein coding capability.

### LINC01050 is a direct transcriptional target of c-Myc

**Fig. 2:**
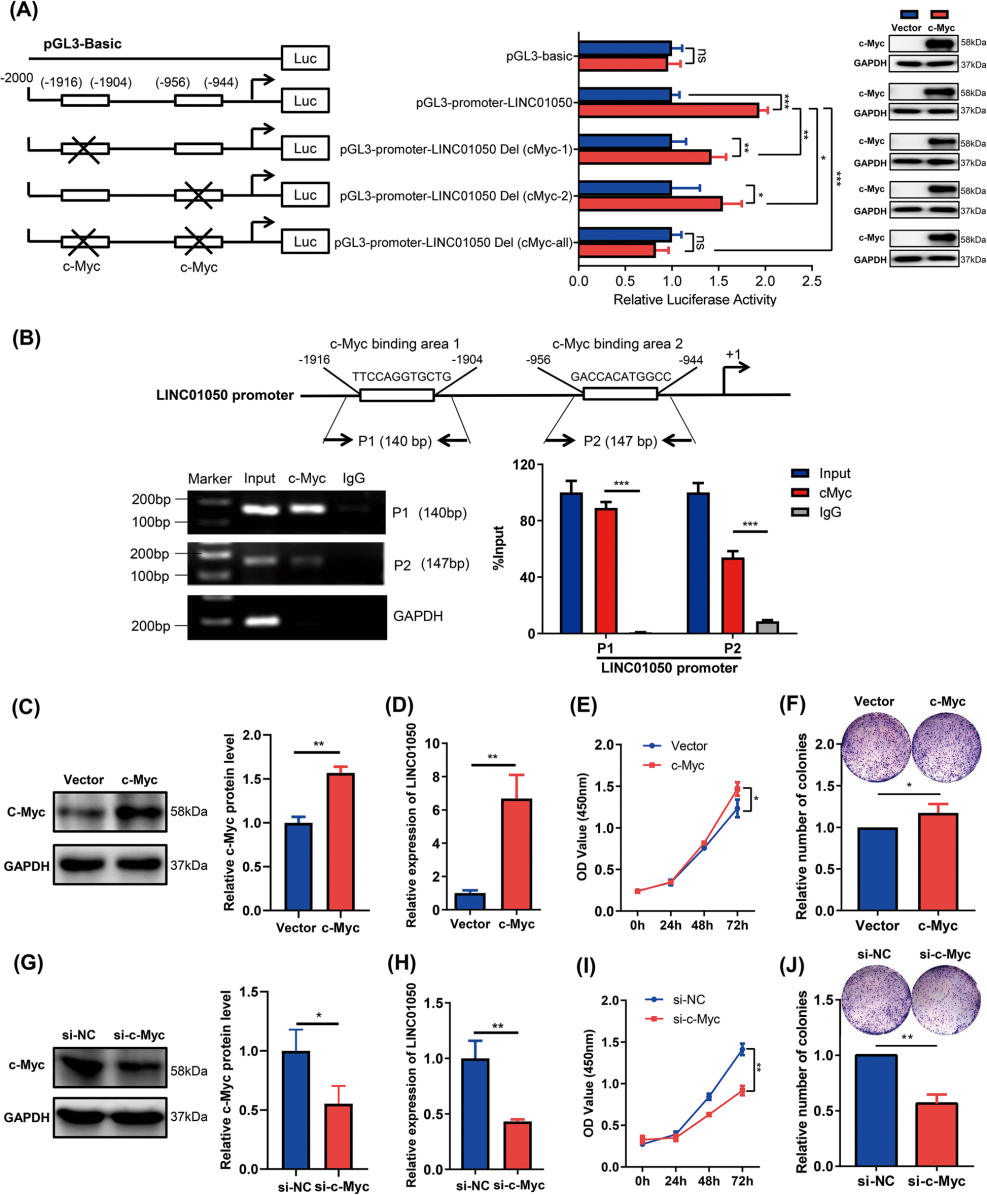
LINC01050 is a direct transcriptional target of c-Myc. **a** Luciferase activity of LINC01050 promoter constructs with a deletion encompassing the c-Myc binding sites in HEK293T cells transfected with pIRES2-vector or pIRES2-c-Myc. Overexpression of c-Myc in HEK293T cells was confirmed by Western blotting. Data are presented as mean ± SD (*n* = 3).^*^P < 0.05; ^**^P < 0.01; ^***^P < 0.001. ns, not significant. **b** ChIP analysis of c-Myc enrichment at the LINC01050 promoter in KATO III cells. The data are represented as the mean ± SD. ^***^P < 0.001. **c** Western blot analysis of c-Myc protein expression in KATO III cells transfected with pIRES2-vector or pIRES2-c-Myc. Data are presented as mean ± SD (*n* = 3). ^**^P < 0.01. **d** qRT-PCR analysis of LINC01050 expression in KATO III cells transfected with pIRES2-vector or pIRES2-c-Myc. Data are presented as mean ± SD (*n* = 3). ^**^P < 0.01. **e** Overexpression of c-Myc promoted KATO III cell growth as revealed by CCK8 assays. Data are presented as mean ± SD (*n* = 3). ^*^P < 0.05. **f** Overexpression of c-Myc in KATO III cells promoted plate colony formation. Data are presented as mean ± SD (*n* = 3). ^*^P < 0.05. **g** Western blot analysis of c-Myc expression in KATO III cells transfected with si-NC or si-c-Myc. Data are presented as mean ± SD (*n* = 3). ^*^P < 0.05. **h** qRT-PCR analysis of LINC01050 expression in KATO III cells transfected with si-NC or si-c-Myc. Data are presented as mean ± SD (*n* = 3). ^**^P < 0.01. **i** Downregulation of c-Myc inhibited KATO III cell growth as revealed by the CCK8 assays. ^**^P < 0.01. **j** Downregulation of c-Myc in KATO III cell inhibited plate colony formation. Data are presented as mean ± SD (*n* = 3). ^**^P < 0.01

Next, we explored the mechanistic significance of high LINC01050 expression in GC. Potential transcription activators of LINC01050 were screened using bioinformatics analysis; namely, the genomic sequence region upstream (~ 2 kb upstream) of the gene coding for LINC01050 was inspected using the UCSC promoter sequence analysis tools. Two putative c-Myc binding sites were found within the promoter region of LINC01050 (Fig. 2a). In the presence of c-Myc, wild-type LINC01050 promoter activity was increased. Meanwhile, individually deleting either c-Myc binding site significantly reduced promoter activity and the simultaneous deletion of bothimpaired promoter activity further (Fig. 2a). These results indicated that the c-Myc binding sites on the LINC01050 promoter might be critical for c-Myc mediated LINC01050 transcription. To further verify the direct binding of c-Myc to the LINC01050 promoter, we performed a ChIP assay with anti-c-Myc. After the immunoprecipitation, the fragments were amplified using primers flanking the consensus c-Myc binding sites in the LINC01050 promoter (Fig. 2b). Amplification products of the expected length were detected from the input DNA, and the DNA fragments were immunoprecipitated using the anti-c-Myc antibody. However, no PCR amplification products were identified when the immunoprecipitation was done with an anti-IgG antibody (Fig. 2b). These results indicated that c-Myc binds to the LINC01050 promoter region.

Next, we examined the influence of c-Myc on LINC01050 expression. The qRT-PCR analysis showed that LINC01050 expression was significantly increased with c-Myc overexpression in KATO III, HGC-27 and BGC823 cells (Fig. 2c-d and Additional file 2: Fig. S1a-b). In addition, the CCK8 and colony formation assays revealed that overexpression of c-Myc promoted GC cell growth (Fig. 2e and f). Meanwhile, knockdown of *c-Myc* by siRNA in the KATO III cells decreased LINC01050 expression (Fig. 2g and h) and inhibited cell growth (Fig. 2i and j) and proliferation (Additional file 2: Fig. S2). In addition, knockdown of LINC01050 by siRNAs in KATO III cells creversed c-Myc-mediated cell proliferation (Additional file 2: Fig. S3). These data suggested that c-Myc positively regulates LINC01050 expression by binding toits promoter.

### Overexpression of LINC01050 promotes GC cell proliferation, migration, invasion, and epithelial-mesenchymal transition (EMT) in vitro and tumor growth in vivo

**Fig. 3:**
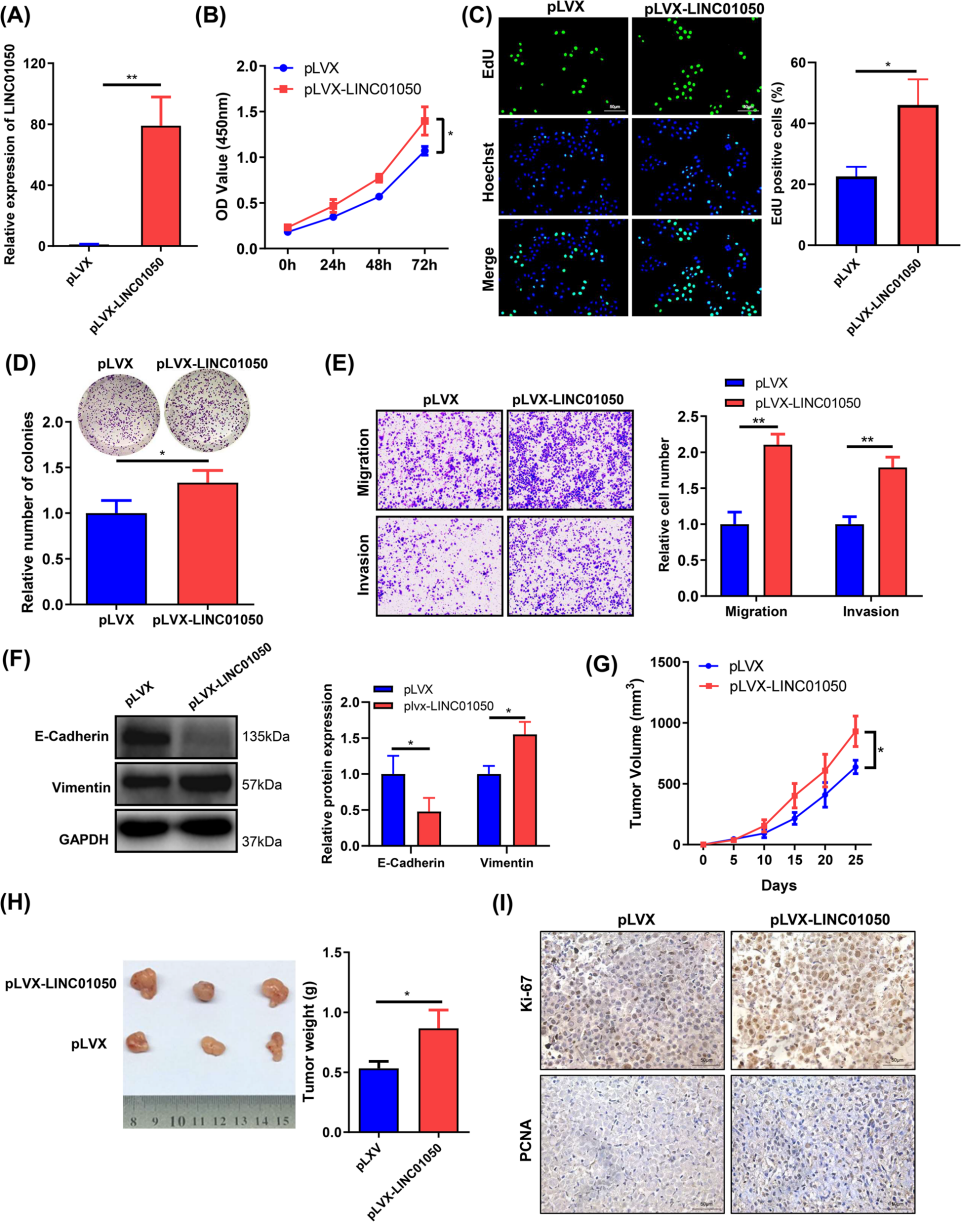
LINC01050 overexpression promotes cell proliferation, metastasis, and EMT in vitro and tumor growth in vivo. **a** qRT-PCR analysis of LINC01050 expression in KATO III cells transduced with pLVX-vector or pLVX-LINC01050. Data are presented as mean ± SD (*n* = 3). ^**^P < 0.01. **b-c** Cell proliferation of KATO III cells transduced with pLVX-vector or pLVX-LINC01050 as determined by CCK8 (**b**) and EdU assays (**c**). Data are presented as mean ± SD (*n* = 3). ^*^P < 0.05. Scale bar = 50 μm. **d** Colony-forming capabilities of KATO III cells transfected with pLVX-vector or pLVX-LINC01050, as determined by plate colony-formation assays. Data are presented as mean ± SD (*n* = 3). ^*^P < 0.05. **e** Migration and invasion abilities of KATO III cells transduced with pLVX-vector or pLVX-LINC01050, as assessed by Transwell assays. Data are presented as mean ± SD (*n* = 3). ^**^P < 0.01. **f** Western blot analysis of EMT-related proteins (E-cadherin and vimentin) in KATO III cells transduced with pLVX-vector or pLVX-LINC01050. GAPDH was used as an internal control. Data are presented as mean ± SD (*n* = 3). ^*^P < 0.05. **g** Growth curves of tumors from KATO III cells transduced with pLVX-vector or pLVX-LINC01050 in tumor-bearing nude mice. Data are presented as mean ± SD (*n* = 5). ^*^P < 0.05. **h** Tumor weights from KATO III cells transduced with pLVX-vector or pLVX-LINC01050 in tumor-bearing nude mice (right panel, *n* = 5). ^*^P < 0.05. The data are presented as the mean ± SD. Three representative images of the tumors from the nude mice are shown (left panel, *n* = 5). **i** Immunohistochemistry to detect the proliferation markers Ki-67 and PTEN in tumor tissue sections. Scale bar = 50 μm

To further explore the role of LINC01050 in GC, we overexpressed it in KATO III cells using a lentiviral vector. Successful overexpression was validated by qRT-PCR (Fig. 3a). The CCK8 and EdU assays revealed that overexpression of LINC01050 promoted cell growth (Fig. 3b-c). In addition, LINC01050 overexpression promoted GES-1 growth (Additional file 2: Fig. S4). The plate colony formation assays revealed that LINC01050 overexpression increased clone survival rate (Fig. 3d). Furthermore, Transwell assays demonstrated that overexpression of LINC01050 significantly promoted GC cell migration and invasion (Fig. 3e).

To examine whether LINC01050 affected the EMT phenotype, we compared the expression of epithelial and mesenchymal markers between control cells and those overexpressing LINC01050. The overexpressing cells exhibited lower levels of the epithelial marker E-cadherin and higher levels of the mesenchymal marker vimentin (Fig. 3f). Moreover, a tumorigenesis study in nude mice revealed that overexpression of LINC01050 promoted tumor growth (Fig. 3g). In parallel, the mean tumor weight at the end of the experiment was higher in the pLVX-LINC01050 group than in the control vector group (Fig. 3h). Ki67 and PCNA staining of the subcutaneous tumor further confirmed that the ectopic expression of LINC01050 promoted GC cell proliferation in vivo (Fig. 3i).

### Knockdown of LINC01050 inhibits GC cell growth in vitro and in vivo

**Fig. 4:**
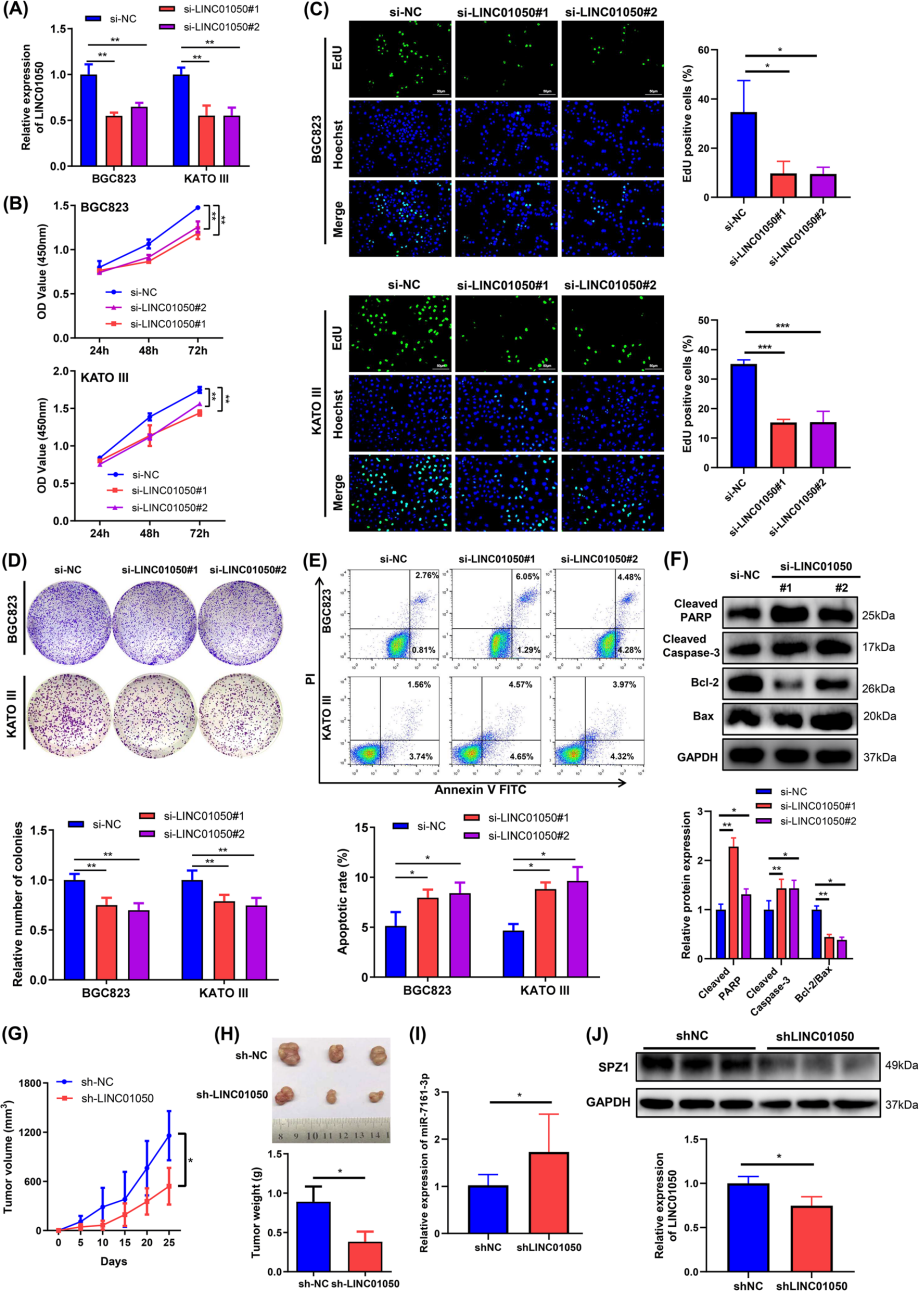
LINC01050 knockdown inhibits gastric cancer cell proliferation and induces apoptosis in vitro and inhibits tumor growth in vivo. **a** qRT-PCR analysis of LINC01050 expression in BGC823 and KATO III cells transfected with si-NC (negative control), si-LINC01050#1, or si-LINC01050#2. Data are presented as mean ± SD (*n* = 3). ^**^P < 0.01. **b-c** Proliferation of BGC823 and KATO III cells transfected with si-NC (negative control), si-LINC01050#1, or si-LINC01050#2, as determined using CCK8 (**b**) and EdU assays (**c**). Data are presented as mean ± SD (*n* = 3). ^**^P < 0.01. **d** Colony-formation capabilities of BGC823 and KATO III cells transfected with si-NC (negative control), si-LINC01050#1, or si-LINC01050#2, as determined using plate colony formation assays. Data are presented as mean ± SD (*n* = 3). ^**^P < 0.01. **e** Cell apoptosis in BGC823 and KATO III cells transfected with si-NC (negative control), si-LINC01050#1, or si-LINC01050#2 for 48 h, analyzed using flow cytometry by Annexin V-FITC and Propidium iodide (PI) staining. Data are presented as mean ± SD (*n* = 3). ^*^P < 0.05. **f** Western blot analysis of cleaved PARP 1, cleaved Caspase-3, Bcl-2, and Bax expression. GAPDH was used as an internal control. Data are presented as mean ± SD (*n* = 3). ^*^P < 0.05, ^**^P < 0.01. **g** Growth curves of tumors from KATO III cells transduced with lentiviral sh-LINC01050 in tumor-bearing nude mice. Data are presented as mean ± SD (*n* = 5). ^*^P < 0.05. **h** Weights of tumors from nude mice. The values are presented as the means ± SD (lower panel, *n* = 5). Three representative images of the tumors from the nude mice are shown (upper panel). ^*^P < 0.05. **i** qRT-PCR analysis of miR-7161-3p expressions in subcutaneous tumor tissues of KATO III cells transduced with lentiviral shNC (negative control) or shLINC01050. Data are presented as mean ± SD (*n* = 3). *P < 0.05. **j** Western blot analysis of SPZ1 expressions in subcutaneous tumor tissues of KATO III cells transduced with lentiviral shNC or shLINC01050. GAPDH was used as an internal control

Both BGC823 and KATO III cells showed high expression of LINC01050 compared to normal cells. Accordingly we knocked down LINC01050 in these cells by transfecting them with the appropriate siRNAs (si-LINC01050#1 and si-LINC01050#2). Knockdown efficiency was validated by qRT-PCR (Fig. 4a) and Northern blot (Additional file 2: Fig. S5). Knockdown of LINC01050 in the BGC823 and KATO III cells by either si-LINC01050#1 or si-LINC01050#2 significantly inhibited proliferation and colony formation (Fig. 4b-d). Moreover, both cell lines exhibited higher apoptotic rates when transfected with the siRNAs (Fig. 4e), indicating that knockdown of LINC01050 induced apoptosis. Western blot analysis of BGC823 cells transfected with either siRNA further revealed significantly increased expression of cleaved PARP 1 and cleaved Caspase-3, along with a decreased Bcl-2/Bax ratio (Fig. 4f). We further established KATO III cells transduced with lentiviral LINC01050 shRNA or shNC and confirmed that knockdown of LINC01050 by sh-LINC01050 inhibited cell growth in vitro (Additional file 2: Fig. S6A and B). Finally, the transduced KATO III cells were inoculated into nude mice to determine whether LINC01050 knockdown affected GC cell growth in vivo. The tumors formed in the sh-LINC01050 group were substantially smaller than those in the control group (Fig. 4g). The mean weight of the xenograft tumors derived from the sh-LINC01050-transfected cells was likewise significantly lower (Fig. 4h). Moreover, the miR-716-3p level was significantly increased, and the SPZ1 protein level was decreased in the subcutaneous xenograft of the shLINC01050-KATO III cells compared to that of shNC-KATO III cells (Fig. 4i and j).

### LINC01050 knockdown inhibits GC cell migration, invasion, and EMT in vitro and lung metastasis in vivo

**Fig. 5:**
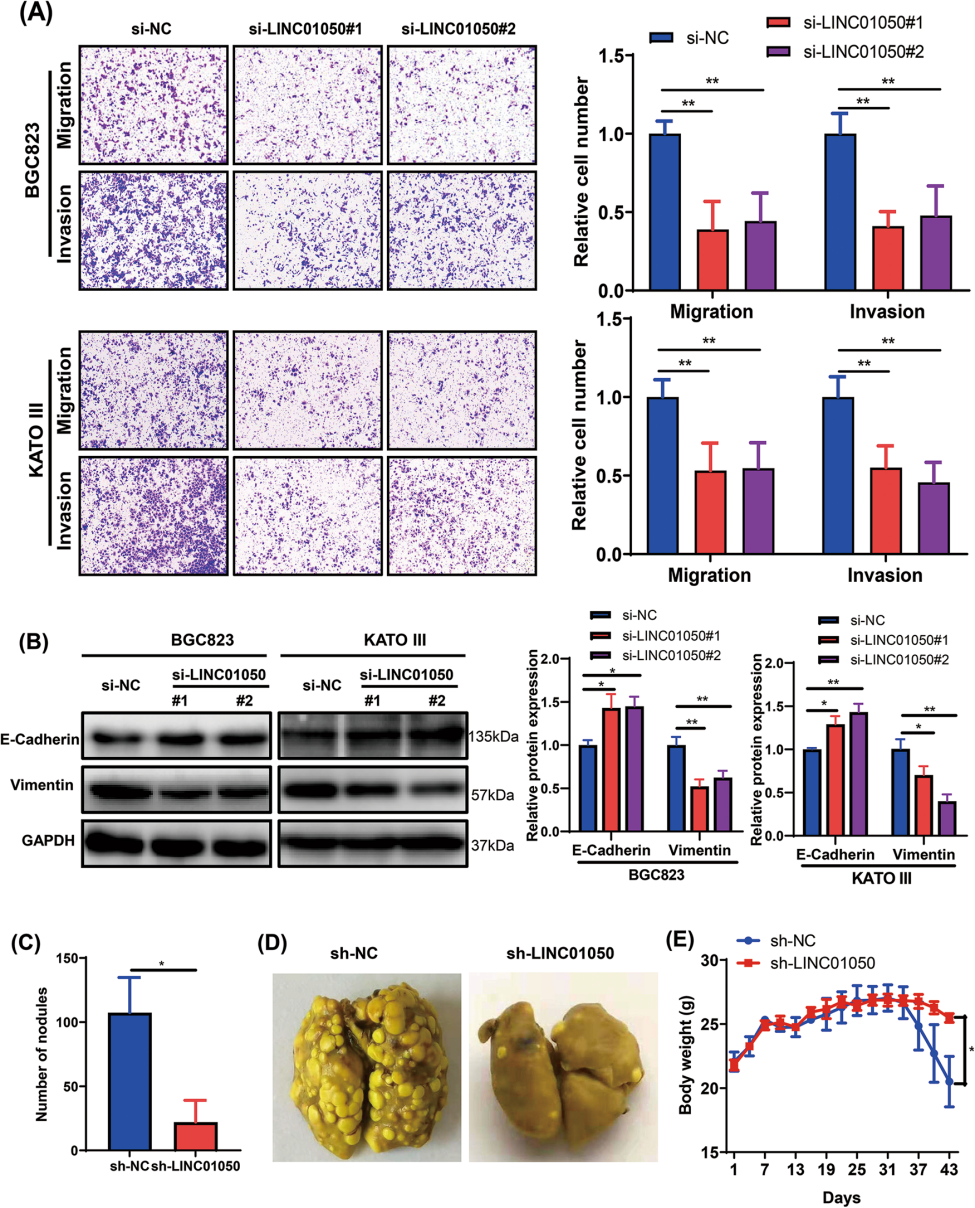
LINC01050 knockdown inhibits gastric cancer cell migration and invasion in vitro and metastasis in vivo. **a** Migration and invasion abilities of BGC823 and KATO III cells transfected with si-NC (negative control), si-LINC01050#1, or si-LINC01050#2, as assessed by Transwell assays. The data are presented as the mean ± SD. ^**^P < 0.01. Scale bar = 100 μm. **b** Western blot analysis of EMT-related protein expression (E-cadherin and vimentin) in BGC823 and KATO III cells transfected with si-NC (negative control), si-LINC01050#1, or si-LINC01050#2. GAPDH was used as an internal control. Data are presented as mean ± SD (*n* = 3). ^*^P < 0.05, ^**^P < 0.01. **c** Statistical quantification of lung metastatic nodules (*n* = 5) produced after BGC823 cells transduced with lentiviral shNC or shLINC01050 were injected into nude mice via the tail vein. The data are represented as the mean ± SD. ^*^P < 0.05. **d** Representative photographs showing the macroscopic appearance of lung metastases. **e** Body weights of mice were recorded after a tail vein injection of the BGC823 cells transduced with lentiviral shNC or shLINC01050. Data are presented as mean ± SD (*n* = 5).^*^P < 0.05

Transwell assays revealed that in the BGC823 and KATO III cells, knockdown of LINC01050 by si-LINC01050#1 and si-LINC01050#2 significantly suppressed cell migration and invasion (Fig. 5a). LINC01050 knockdown also increased the level of E-cadherin and decreased that of vimentin (Fig. 5b). Moreover, knockdown of LINC01050 by shRNA inhibited BGC823 cell migration and invasion (Additional file 2: Fig. S7). To verify the effect of LINC01050 knockdown on tumor metastasis in vivo, BGC823 cells transduced with lentiviral sh-LINC01050 or shNC were injected into the tail veins of nude mice. The number of lung metastases in the sh-LINC01050 treatment group was significantly lower than in the control group (Fig. 5c and d). At 43 days post-injection, the mouse body weights in the sh-LINC01050 treatment group were significantly greater (Fig. 5e).

### LINC01050 binds to miR-7161-3p which targets SPZ1

**Fig. 6:**
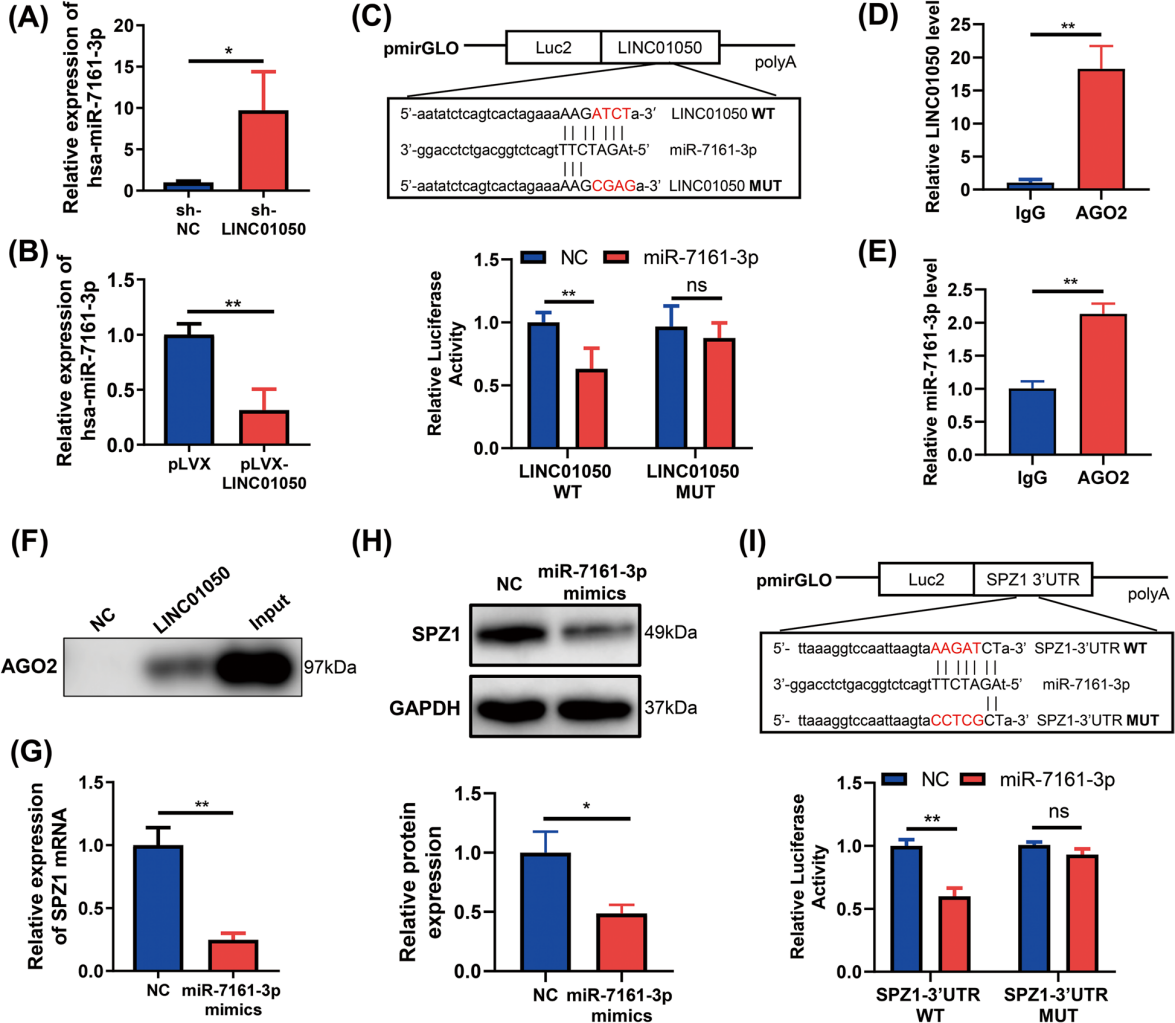
LINC01050 directly binds to miR-7161-3p which targets SPZ1 by binding to its 3’UTR. **a** qRT-PCR analysis of miR-7161-3p expression in KATO III cells transduced with lentiviral control shRNA (sh-NC) or sh-LINC01050. ^*^P < 0.05. **b** qRT-PCR analysis of miR-7161-3p expression in KATO III cells transduced with pLVX vector or pLVX-LINC01050. ^**^P < 0.01. **c** Diagram of the luciferase reporter vectors containing the wild-type (WT) or mutant (MUT) LINC01050 sequences, with the highly conversed putative miR-7161-3p binding sites indicated. In the HEK293T cells, the miR-7161-3p mimic reduced the luciferase activity of the WT reporter relative to the negative control, but had little impact on the MUT reporter activity. ^**^P < 0.01; ns, not significant. **d-e** Detection of LINC01050 and miR-7161-3p by qRT-PR in immunoprecipitated RNA after performing an anti-AGO2 RIP in KATO III cells. IgG was the negative control. ^**^ *P* < 0.01. **f** Enrichment of AGO2 protein in pull-down assay performed using LINC01050 or a negative control (NC) incubated with cell extracts. **g** qRT-PCR analysis of *SPZ1* mRNA expression in KATO III cells transfected with the NC or miR-7161-3p mimics. ^**^P < 0.01. **h** Western blot analysis of SPZ1 protein expression in KATO III cells transfected with the NC or miR-7161-3p mimics. *P < 0.05. **i** Diagram of the luciferase reporter vectors containing the WT or MUT sequence of the SPZ1 3’UTR, with the highly conversed putative miR-7161-3p binding sites indicated. Luciferase activity of pmirGLO vectors containing the WT or MUT *SPZ1* 3’UTR sequence after co-transfection into HEK293T cells with the NC or miR-7161-3p mimics. ^**^P < 0.01; ns, not significant

LncRNAs are reported to regulate target gene expression by interacting with RNA-binding proteins, such as PRC2, or by acting as a molecular sponge for miRNAs [24]. Our results showed that LINC01050 was both located in the cytoplasm and nucleus, suggesting that it might partly regulate target expression at the posttranscriptional level. Thus, we hypothesized that there might be an interaction between LINC01050 and miRNAs in the context of GC. We utilized online software (LncBase Predicted v.2) to search for miRNAs showing complementary base pairing with LINC01050 and observed potential binding sites for miR-7161-3p. Knockdown of LINC01050 in KATO III cells increased the expression of miR-7161-3p (Fig. 6a), while its overexpression significantly decreased miR-7161-3p expression (Fig. 6b). To further determine whether LINC01050 acts as a miR-7161-3p “sponge”, wild-type (WT) and mutated (MUT) miR-7161-3p binding sequences were used to construct luciferase reporter vectors (Fig. 6c). Subsequent dual-luciferase reporter assays in HEK293T cells revealed that miR-7161-3p significantly suppressed the luciferase activity associated with the LINC01050 WT reporter, but not the LINC01050 MUT reporter (Fig. 6c).

The RNA-binding protein Argonaute 2 (AGO2) is a critical component of the RNA-induced silencing complex (RISC) and exerts a pivotal role in miRNA functions [25]. Accordingly, we conducted anti-AGO2 RIP and RNA pull-down experiments. The RIP experiment confirmed that the anti-AGO2 group was enriched for both LINC01050 and miR-7161-3p (Fig. 6d and e). Futhermore, the RNA pull-down experiment showed significant enrichment of AGO2 in the presence of LINC01050 compared with the negative control (Fig. 6f). The RNA pull-down assay also revealed that miR-7161-3p was enriched by the LINC01050 biotin-labeled probe (Additional file 2: Fig. S8). Moreover, the relationship between LINC01050 and miR-7161-3p expression was analyzed in 29 GC tissues by qRT-PCR. The results showed a negative correlation between LINC01050 and miR-7161-3p expression in GC tissues (Additional file 2: Fig. S9).

Utilizing the software TargetScan, we found that SPZ1 might be a possible target of miR-7161-3p. To confirm this relationship, we assessed SPZ1 mRNA and protein levels in KATO III cells transfected with miR-7161-3p, and found that both were significantly decreased (Fig. 6g-h). Next, a fragment of the SPZ1 3’UTR containing the predicted miR-7161-3p binding site (SPZ1-3’UTR WT) and a mutated version lacking the site (SPZ1-3’UTR MUT) were cloned into luciferase reporter vectors (Fig. 6i). Upon transfection of the vectors into HEK293T cells together with control miRNA or the miR-7161-3p mimic, the miR-7161-3p mimic remarkably suppressed the luciferase activity associated with SPZ1-3’UTR WT but not the SPZ1-3’UTR MUT (Fig. 6i). Moreover, treating the cells with miR-7161-3p inhibitors promoted cell proliferation, and this was partially reversed by co-transfection with si-SPZ1(Additional file 2: Fig. S10A-C).

### LINC01050 modulates GC cell proliferation, migration, invasion, and EMT by regulating the miR-7161-3p/SPZ1 axis

**Fig. 7:**
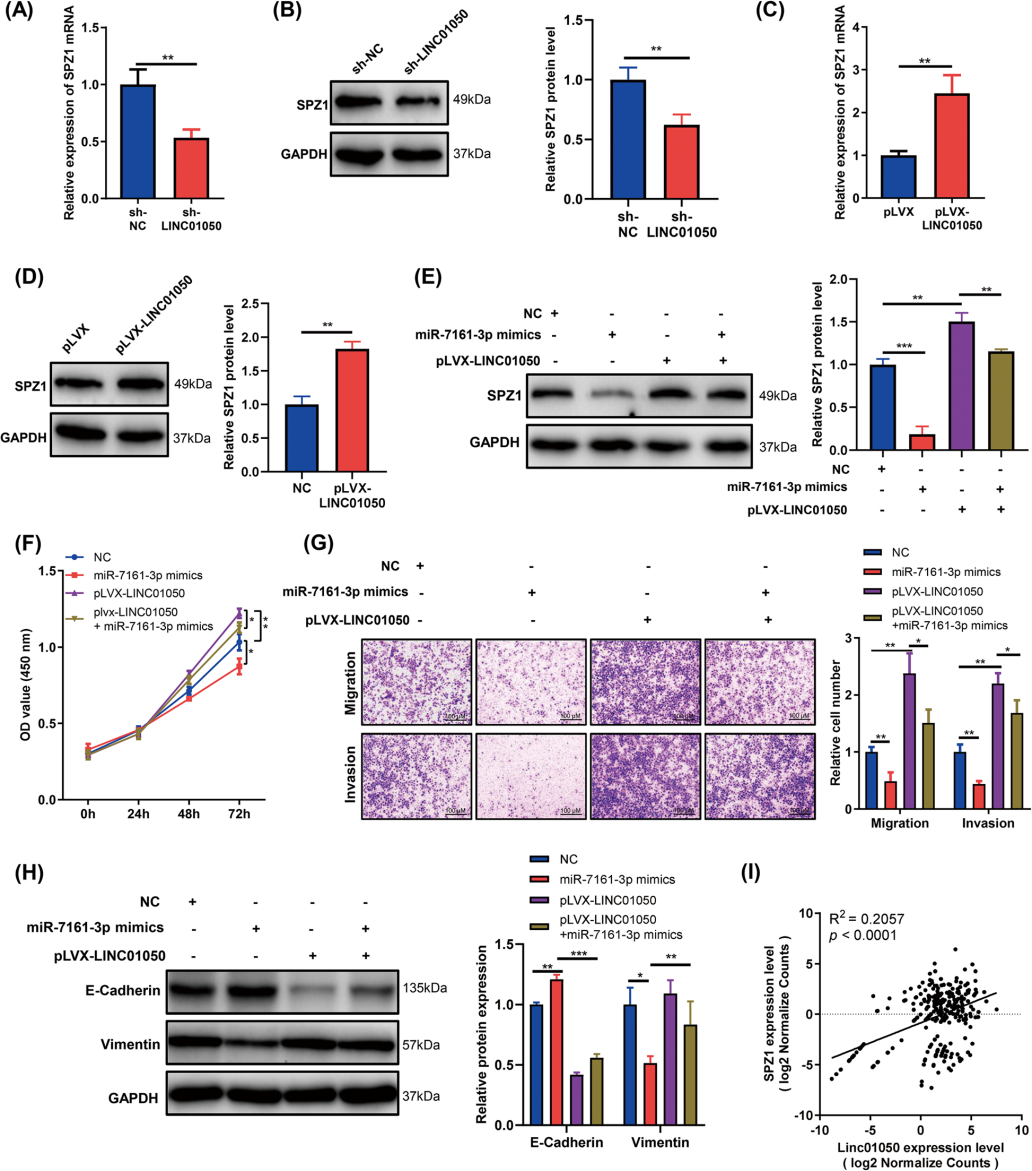
LINC01050 promotes gastric cancer cell proliferation, migration, invasion, and EMT by regulating the miR-7161-3p/*SPZ1* axis. **a** qRT-PCR analysis of *SPZ1* mRNA expression in KATO III cells transfected with the control shRNA (sh-NC) or sh-LINC01050. ^**^P < 0.01. **b** Western blot analysis of SPZ1 protein level in KATO III cells stably expressing the sh-NC or sh-LINC01050. ^**^P < 0.01. **c** qRT-PCR analysis of SPZ1 mRNA expression in KATO III stable cell line with the pLVX vector or overexpressing pLVX-LINC01050. ^**^P < 0.01. **d** Western blot analysis of SPZ1 protein level in KATO III cells transduced with the pLVX vector or pLVX-LINC01050. ^**^P < 0.01. **e** Western blot analysis of SPZ1 protein level in KATO III cells transfected with NC, miR-7161-3p mimics, pLVX-LINC01050 or pLVX-LINC01050 plus miR-7161-3p mimics. ^**^P < 0.01, ^***^P < 0.001. **f** Growth curves of KATO III cells transfected with NC, miR-7161-3p mimics, pLVX-LINC01050 or pLVX-LINC01050 plus miR-7161-3p mimics, as revealed using CCK8 assays. ^*^P < 0.05, ^**^P < 0.01. **g** Migration and invasion capabilities of KATO III cells transfected with NC, miR-7161-3p mimics, pLVX-LINC01050 or pLVX-LINC01050 plus miR-7161-3p mimics, as revealed using Transwell assays. ^*^P < 0.05, ^**^P < 0.01. Scale bar = 100 μm. **h** Western blot analysis of EMT-related protein expression (E-cadherin and vimentin) in KATO III cells transfected with NC, miR-7161-3p mimics, pLVX-LINC01050 or pLVX-LINC01050 plus miR-7161-3p mimics. GAPDH protein was used as an internal control. ^*^P < 0.05, ^**^P < 0.01, ^***^P < 0.001. **i** Association of LINC01050 and *SPZ1* expression levels. *P* < 0.0001

Subsequently, we asked whether LINC01050 exerted its function through the miR-7161-3p/SPZ1 axis. Knockdown of LINC01050 in KATO III cells by shRNA significantly decreased SPZ1 mRNA and protein levels (Fig. 7a-b), whereas its overexpression significantly increased SPZ1 mRNA and protein levels (Fig. 7c-d). To determine whether miR-7161-3p plays a role in the relationship between LINC01050 and SPZ1, KATO III cells were co-transfected with pLVX-LINC01050 and the miR-7161-3p mimic. Indeed, the increase in SPZ1 protein induced by LINC01050 was effectively reversed by miR-7161-3p (Fig. 7e), and the reduction of SPZ1 associated with LINC01050 knockdown was reversed by miR-7161-3p inhibitors (Additional file 2: Fig. S11a-c). Notably, the pLVX-LINC01050-mediated promotion of cell proliferation, migration, and invasion was partially rescued by co-transfection with the miR-7161-3p mimic (Fig. 7f-g), as was LINC01050-induced EMT (decreased E-cadherin expression and increased vimentin expression) (Fig. 7h). Moreover, we analyzed the correlation between LINC01050 and SPZ1 expression utilizing TCGA data and identified a positive correlation consistent with the existence of a LINC01050/miR-7161-3p/*SPZ1* regulatory axis (Fig. 7i).

### *SPZ1* knockdown inhibits GC cell growth, migration, invasion, and EMT

**Fig. 8:**
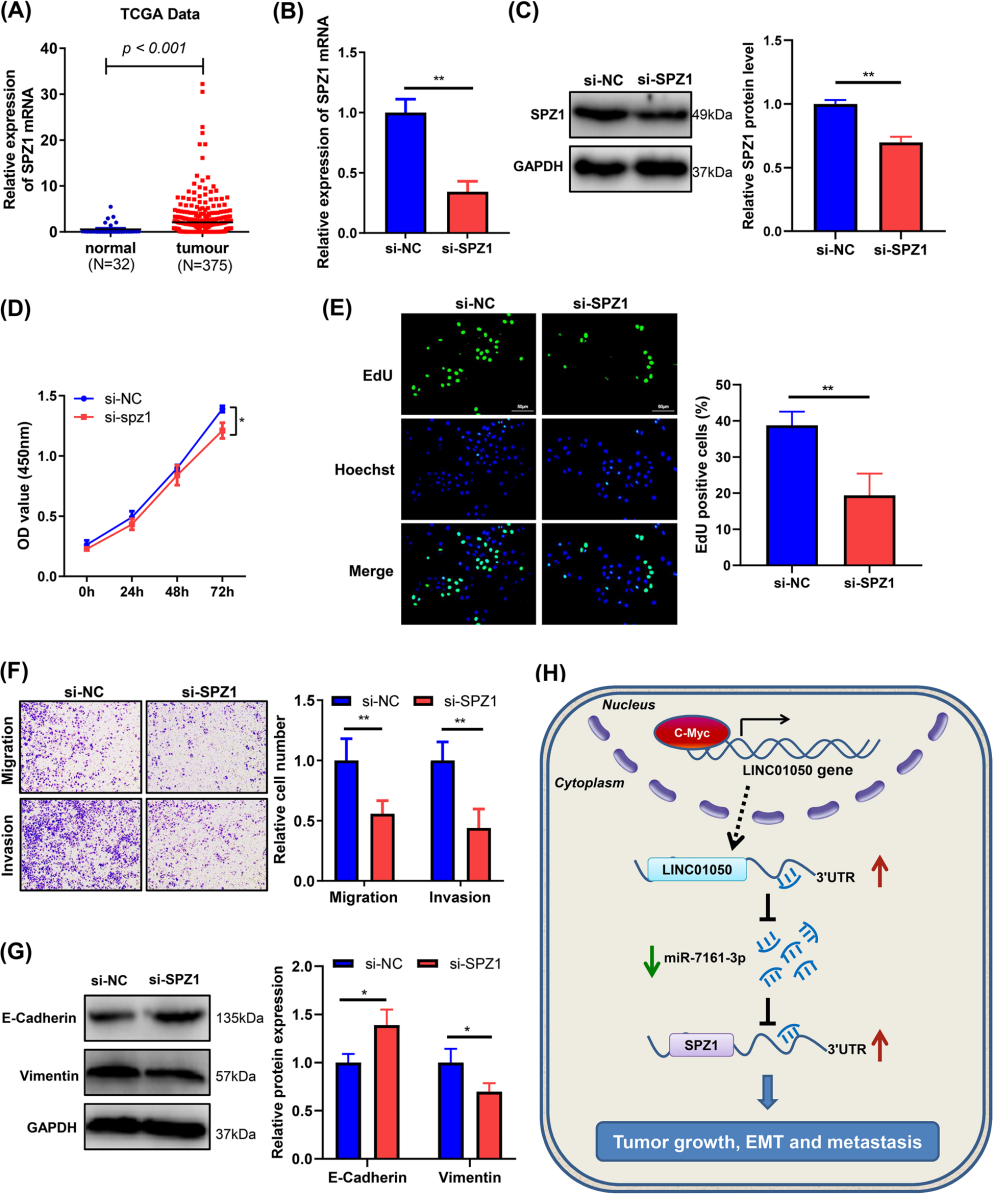
*SPZ1* knockdown inhibits gastric cancer cell proliferation, migration, invasion, and EMT. **a** Relative expression of *SPZ1* mRNA in gastric cancer tissues and normal tissues according to TCGA data (*P* < 0.001). **b** *SPZ1* mRNA levels in KATO III cells transfected with si-NC or si-SPZ1, as determined by qRT-PCR. Data are presented as mean ± SD (*n* = 3). ^**^P < 0.01. **c** SPZ1 protein expression in KATO III cells transfected with si-NC or si-SPZ1. Data are presented as mean ± SD (*n* = 3). ^**^P < 0.01. **d-e** Proliferation of KATO III cells transfected with si-NC (negative control) or si-SPZ1, as determined using CCK8 (**d**) and EdU (**e**) assays. Data are presented as mean ± SD (*n* = 3). ^*^P < 0.05, ^**^P < 0.01. Scale bar = 50 μm. **f** Migration and invasion capabilities of KATO III cells transfected with si-NC or si-SPZ1, revealed using Transwell assays. The data are represented as the mean ± SD (*n* = 3). ^**^P < 0.01. **g** Western blot analysis ofEMT-related protein expression (E-cadherin and vimentin) in KATO III cells transfected with si-NC or si-SPZ1. Data are presented as mean ± SD (*n* = 3). ^*^P < 0.05. **h** A proposed model illustrating the regulatory role of c-Myc-activated lncRNA LINC01050 in promoting gastric cancer growth and metastasis by sponging miR-7161-3p to regulate SPZ1 expression

Analysis of TCGA data revealed that *SPZ1* is increased in GC tissues compared with normal tissues (Fig. 8a). To investigate the role of SPZ1 in GC, its expression in KATO III cells was knocked down by siRNA. The efficacy of the knockdown was confirmed by qRT-PCR and Western blot analyses (Fig. 8b-c). *SPZ1* knockdown reduced cell growth (Fig. 8d-e), inhibited migration and invasion (Fig. 8f), and reversed the phenotype induced by LINC01050 in GC cells (Additional file 2: Fig. S12a-c). Western blot analysis also revealed that the knockdown of SPZ1 suppressed EMT (i.e., it increased the level of E-cadherin protein and decreased the level of vimentin protein) (Fig. 8g).

## Discussion

Recently, growing evidence has revealed that the newly discovered lncRNAs play pivotal roles in human diseases, especially cancer. The oncogene c-Myc is often deregulated in human cancers and contributes to tumor progression [26]. As a transcriptional factor, c-Myc is involved in many biological processes, such as metabolism, cell growth, cell cycle regulation, and apoptosis [27]. It targets many protein-coding genes. In addition, many lncRNAs are newly-proven downstream targets of c-Myc [28–33], and play essential roles in cancer cell proliferation and tumorigenesis [33–35]. Lu et al. reported the c-Myc-targeted lncRNA DANCR was overexpressed in various tumor types and promoted cancer cell proliferation [35]. In addition, the c-Myc-induced lncRNA, LncRNA-MIF, plays an important role in c-Myc-mediated aerobic glycolysis [33]. Cao et al. likewise identified a novel c-Myc-induced lncRNA, LAST, which interacts with CNBP to promote the stability of *CCND1* mRNA [36].

In this study, we identified LINC01050 as a novel c-Myc-activated lncRNA that functions as a molecular sponge to absorb cytosolic miR-7161-3p, thereby reducing the miR-7161-3p-mediated translational repression of *SPZ1*, which contributes to GC progression (Fig. 8h). However, no significant association between c-Myc and LINC01050 expression in the context of GC was identified based on TCGA data (Additional file 2: Fig. S13), suggesting that LINC01050 expression may be regulated in a more complex manner, not just by c-Myc alone. To date, the biological function and expression pattern of LINC01050 in cancer have not been unraveled. We found that LINC01050 was up-regulated in GC tissues and cell lines, and its high expression in GC patients was positively correlated with a poor prognosis. Furthermore, LINC01050 overexpression promoted GC cell proliferation, migration, invasion, and EMT in vitro and tumor growth in vivo. At the same time, its knockdown inhibited GC cell proliferation, migration, invasion, and EMT in vitro, as well as tumor growth and metastasis in vivo*.* These results indicate that LINC01050 might play a vital role in GC progression.

The ceRNA theory indicates that lncRNAs function as sponges for miRNAs and thereby regulate the expression of coding genes [37, 38]. For example, the novel lncRNA, MCM3AP-AS1, promotes the growth of hepatocellular carcinoma by acting as a ceRNA for miR-194-5p [39]. In addition, the lncRNA LINC01234 promotes the growth of gastric cancer by acting as a ceRNA for miR-204-5p [40]. We found that LINC01050 localized to the cytoplasm and nucleus, suggesting that it may partly function as an endogenous miRNA sponge. Bioinformatics analyses and luciferase reporter assays revealed that miR-7161-3p was a target of LINC01050. miR-7161-3p overexpression was found to inhibit GC cell growth, migration, invasion, and EMT. Furthermore, rescue experiments revealed that overexpression of miR-7161-3p partly reverse the growth-promoting effect induced by LINC01050, indicating that LINC01050 promots GC progression, at least in part, through the suppression of miR-7161-3p activity.

In regulation effected by the ceRNA network, miRNA targets are integral. Using the TargetScan database, we identified SPZ1 as a potential miR-7161-3p target. SPZ1 is up-regulated in various human cancers and functions as a tumor promoter [41, 42]. For example, Wang LT and colleagues found that SPZ1 promoted EMT and metastasis in liver cancer [21, 43], specifically by trans-activating TWIST1, which encodes a master regulator of EMT [43]. In addition, SPZ1 homodimers activate TWIST1 expression and are acetylated by TIP60 to form a heterodimeric SPZ1-TWIST1 complex, which promotes EMT and initiates tumor metastasis [44]. Moreover, SPZ1 overexpression in breast cancer promotes drug-resistance and metastases [45]. To confirm that SPZ1 is a direct target of miR-7161-3p, we conducted luciferase reporter assays and verified that miR-7161-3p targetsits 3′UTR. Overexpression of miR-7161-3p in GC cells suppressed SPZ1 mRNA and protein expression. In addition, we found that LINC01050 regulates *SPZ1* expression through its interaction with miR-7161-3p. There was also a positive correlation between LINC01050 and *SPZ1* expression in GC tissues, and analysis of TCGA data revealed that *SPZ1* mRNA was significantly up-regulated in GC. Finally, knockdown of *SPZ1* by siRNA inhibited GC cell proliferation, migration, invasion, and EMT. Together, these results suggest that LINC01050 modulates GC cell proliferation, migration, invasion, and EMT by regulating the miR-7161-3p/*SPZ1* axis.

## Conclusions

In summary, our results demonstrated that LINC01050 is regulated by c-Myc and promotes GC progression by sponging miR-7161-3p to regulate SPZ1 expression. Our findings revealed a novel LINC01050/miR-7161-3p/*SPZ1* axis in GC, and LINC01050 may represent a potential therapeutic target.

### Supplementary Information

**Additional file 1.**

**Additional file 2.**

## Data Availability

The datasets used and/or analysed during the current study are available from the corresponding author on reasonable request. Publicly available data was obtained from the TCGA database (https://portal.gdc.cancer.gov/) and the Ensembl database (https://asia.ensembl.org).

## Abbreviations

AGO2: Argonaute 2
CCK8: Cell Counting Kit-8
ceRNA: Competing endogenous RNA
ChIP: Chromatin immunoprecipitation
DMEM: Dulbecco’s Modified Eagle’s Medium
EMT: Epithelial-mesenchymal transition
FBS: Fetal bovine serum
GC: Gastric cancer
FISH: Fluorescence in situ hybridization
lncRNAs: Long non-coding RNAs
MUT: Mutated
NC: Negative control
ncRNAs: Non-coding RNAs
PI: Propidium iodide
qRT-PCR: Quantitative reverse transcription polymerase chain reaction
RIP: Immunoprecipitation
RISC: RNA-induced silencing complex
STAD: Stomach adenocarcinoma
SD: Standard deviation
TCGA: The Cancer Genome Atlas
WT: Wild-type
