# Correction: c-Myc-activated long non-coding RNA LINC01050 promotes gastric cancer growth and metastasis by sponging miR-7161-3p to regulate SPZ1 expression

**DOI:** 10.1186/s13046-024-02948-6

**Published:** 2024-02-05

**Authors:** Ziwei Ji, Tianbin Tang, Mengxia Chen, Buyuan Dong, Wenjing Sun, Nan Wu, Hao Chen, Qian Feng, Xingyi Yang, Rong Jin, Lei Jiang

**Affiliations:** 1 Department of Gastroenterology, the First Affiliated Hospital of Wenzhou Medical University, Wenzhou, 325000 China; 2 Central Laboratory, the First Affiliated Hospital of Wenzhou Medical University, Wenzhou, 325000 China

**Correction:** *J Exp Clin Cancer Res* **40, 351 (2021)**

**https://doi.org/10.1186/s13046-021-02155-7**

Following the publication of the original article [1], the authors identified a minor error in image-typesetting in Fig. 5a; specifically, the lower panels in Fig. 5a, the incorrect representative image used for invasion transwell assays of KATO III cells transfected with si-LINC01050#1.

**Fig. 5:**
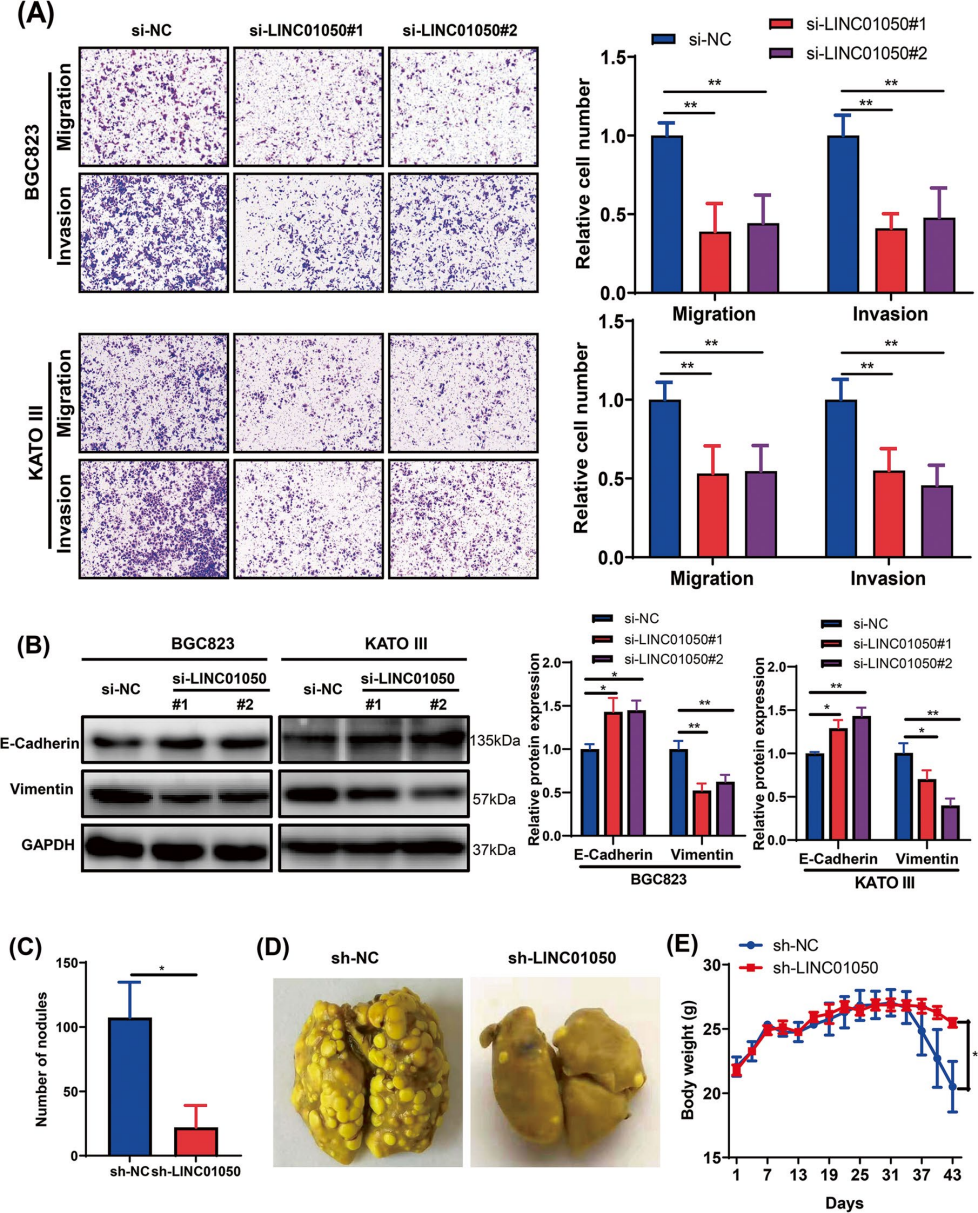
LINC01050 knockdown inhibits gastric cancer cell migration and invasion in vitro and metastasis in vivo. **a** Migration and invasion abilities of BGC823 and KATO III cells transfected with si-NC (negative control), si-LINC01050#1, or si-LINC01050#2, as assessed by Transwell assays. The data are presented as the mean ± SD. ^**^P < 0.01. Scale bar = 100 μm. **b** Western blot analysis of EMT-related protein expression (E-cadherin and vimentin) in BGC823 and KATO III cells transfected with si-NC (negative control), si-LINC01050#1, or si-LINC01050#2. GAPDH was used as an internal control. Data are presented as mean ± SD (*n* = 3). ^*^P < 0.05, ^**^P < 0.01. **c** Statistical quantification of lung metastatic nodules (*n* = 5) produced after BGC823 cells transduced with lentiviral shNC or shLINC01050 were injected into nude mice via the tail vein. The data are represented as the mean ± SD. ^*^P < 0.05. **d** Representative photographs showing the macroscopic appearance of lung metastases. **e** Body weights of mice were recorded after a tail vein injection of the BGC823 cells transduced with lentiviral shNC or shLINC01050. Data are presented as mean ± SD (*n* = 5).^*^P < 0.05

The corrected figure is given below. The correction does not have any effect on the final conclusions of the paper.
