# Supplementary material for: C-Myc-activated long non-coding RNA LINC01050 promotes gastric cancer growth and metastasis by sponging miR-7161-3p to regulate SPZ1 expression
Source: J Exp Clin Cancer Res. 2021 Nov 8;40:351. doi: 10.1186/s13046-021-02155-7 (PMC8573944; doi:10.1186/s13046-021-02155-7)
Supplement: Supplementary file 1 — Additional file 1. [file 13046_2021_2155_MOESM1_ESM.docx]

**Supplementary Tables**

**Table S1** siRNAs target sequences

| **siRNA** | **Target sequence (5’-3’)** |
| --- | --- |
| si-LINC01050 #1 | 5’-CTCACTACTTAGCCCAGAT-3’ |
| si-LINC01050 #2 | 5’-CCAAGCACTTCACAACACT-3’ |
| shRNA-LINC01050 | 5’-CTCACTACTTAGCCCAGAT-3’ |
| si-SPZ1 | 5’-CCATCAAGTTACAGAACAA-3’ |
| si-cMyc | 5’-CGATGTTGTTTCTGTGGAA-3’ |
| si-NC (negative control) | 5’- GGCTCTAGAAAAGCCTATGC -3’ |

**Table S2** Primers for quantitative polymerase chain reaction.

| Gene name | Direction | Primer sequence |
| --- | --- | --- |
| LINC01050 | Forward | 5’-GACGTAACTGCTTCAGGGCA-3’ |
|  | Reverse | 5’-CTTGGCTTTGTCCTTCTCGC-3’ |
| SPZ1 | Forward | 5’-ACAGGAAGCAGAACACTGGAG-3’ |
|  | Reverse | 5’-CAGCTCATGCTTAGCCGACA-3’ |
| GAPDH | Forward | 5’-CCAGCCGAGCCACATCGCTC-3’ |
|  | Reverse | 5’-ATGAGCCCCAGCCTTCTCCAT-3’ |
| miR-7161-3p | RT primer | 5’-GTCGTATCCAGTGCAGGGTCCGAGGTATTCGCACTGGATACGACCCTGGA-3’ |
|  | Forward | 5’-CGTAGATCTTTGACTCTGGCAGTC-3’ |
|  | Reverse | 5’-AGTGCAGGGTCCGAGGTATT-3’ |
| U6 | RT- primer | 5’-AACGCTTCACGAATTTGCGT-3’ |
|  | Forward | 5’-CTCGCTTCGGCAGCACA-3’ |
|  | Reverse | 5’-AACGCTTCACGAATTTGCGT-3’ |

**Table S3** Primers for Chromatin immunoprecipitation (ChIP) assays

| **Gene name** | **Direction** | **Sequence** |
| --- | --- | --- |
| LINC01050 p1 | Forward | 5’-ATGCACCAGTCAGTAGCAAG-3’ |
|  | Reverse | 5’-ACCAGGACTGGTGTCTTTATTGT-3’ |
| LINC01050 p2 | Forward | 5’-AGTGTGGAAGGCTGTAGGGG-3’ |
|  | Reverse | 5’-AAGCCACCTGTTCACTGTGT-3’ |
