# Supplementary material for: C-Myc-activated long non-coding RNA LINC01050 promotes gastric cancer growth and metastasis by sponging miR-7161-3p to regulate SPZ1 expression
Source: J Exp Clin Cancer Res. 2021 Nov 8;40:351. doi: 10.1186/s13046-021-02155-7 (PMC8573944; doi:10.1186/s13046-021-02155-7)
Supplement: Supplementary file 2 — Additional file 2. [file 13046_2021_2155_MOESM2_ESM.docx]

**Supplementary Data**


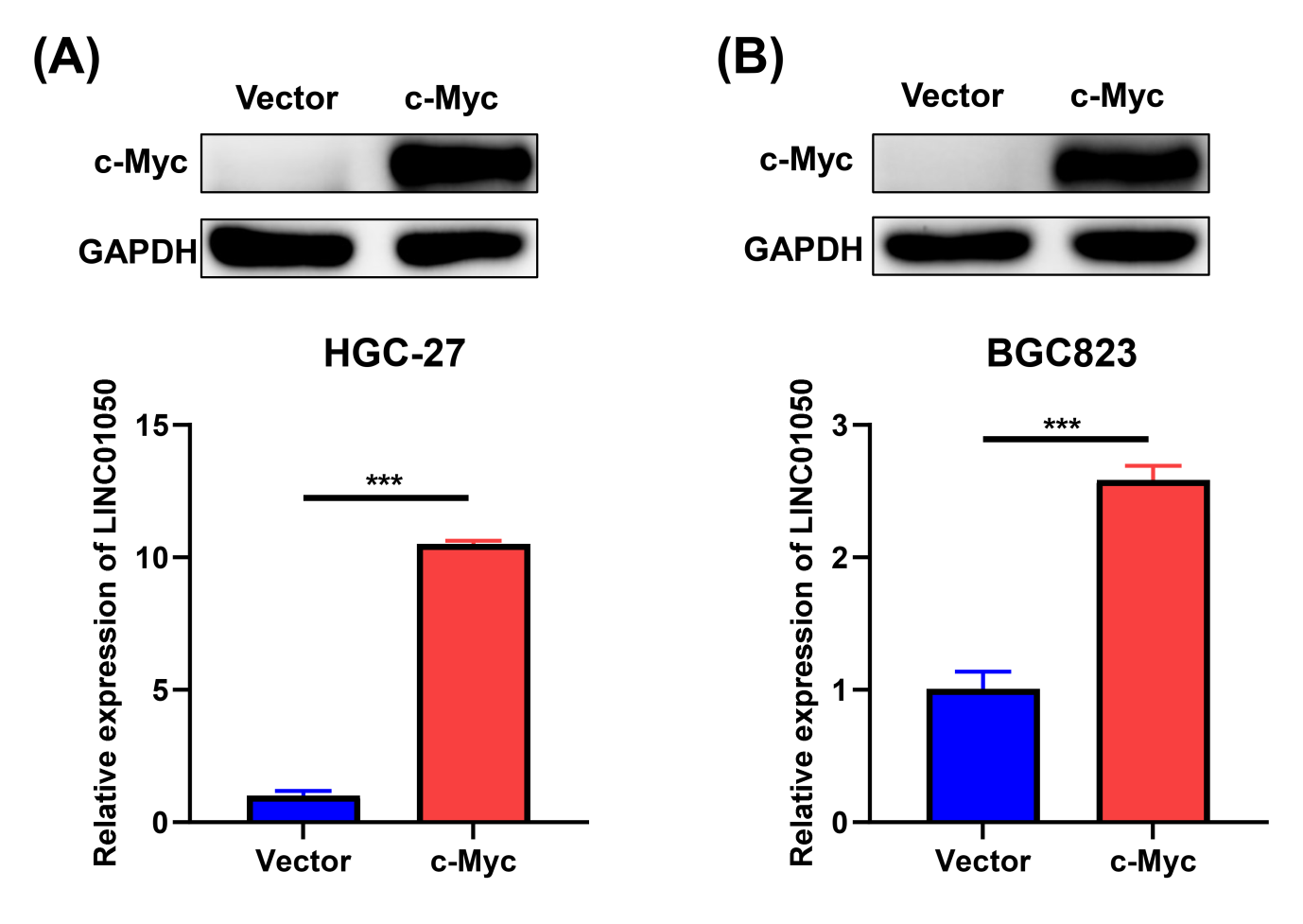


**Figure S1** qRT-PCR analysis of LINC01050 expression in HGC-27 (**a**) and BGC823 (**b**) cells transfected with pIRES2-vector or pIRES2-c-Myc. The transfection efficiency of c-Myc was verified by Western blotting. Data are presented as mean ± SD (n = 3). ****P* < 0.001.

**Figure S2** Knockdown of c-Myc by siRNA inhibited KATO III cell proliferation as revealed by EdU assays. Data are presented as mean ± SD (n = 3). **P* < 0.05.


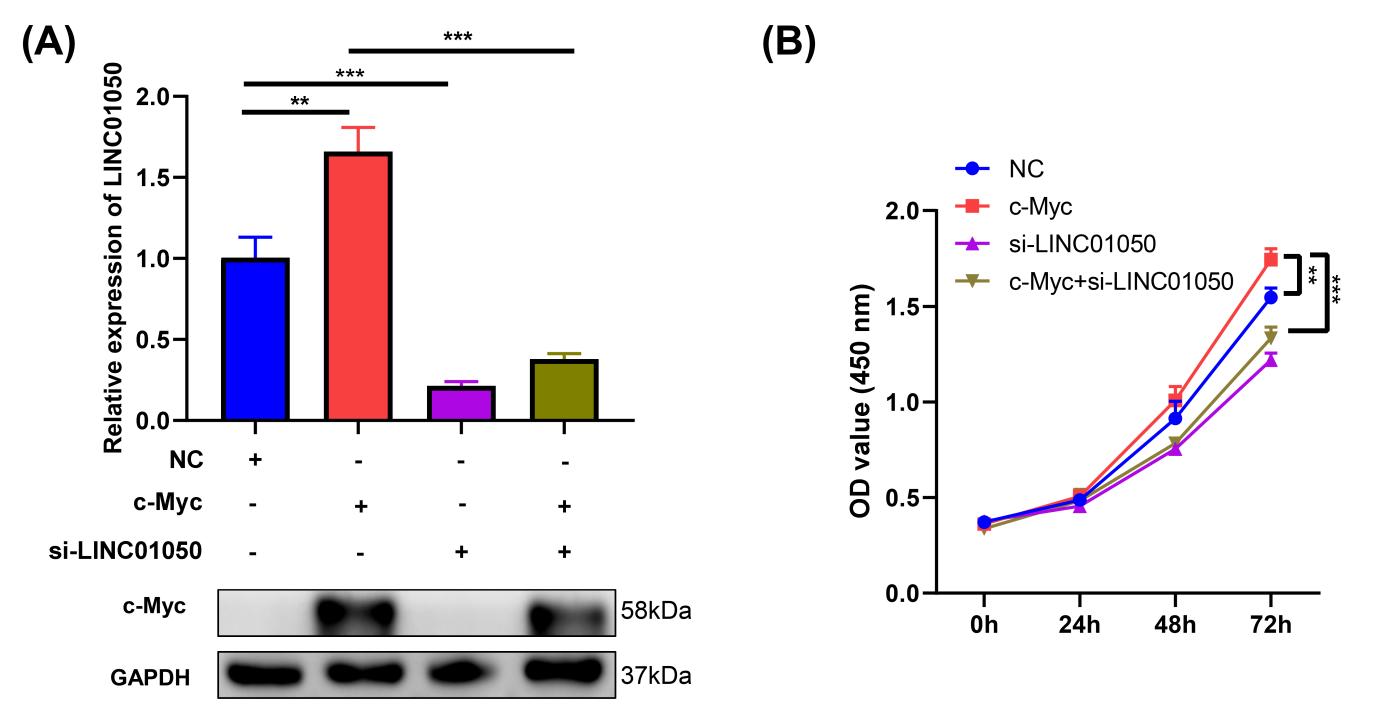


**Figure S3** Knockdown of LINC01050 reverses the c-Myc-mediated cell proliferation in gastric cancer cell line KATO III. **a** LINC01050 and c-Myc expressions were determined by qPCR or Western blotting. **b** Cell growth was assessed by CCK-8 assays. Data are presented as mean ± SD (n = 3). ***P* < 0.01, ****P* < 0.001.

**
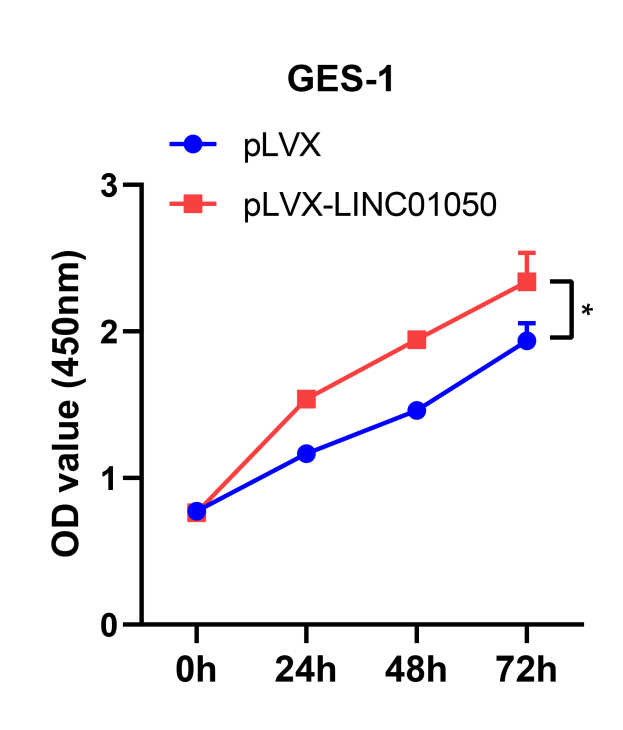
**

**Figure S4** Cell growth of GES-1 cells transfected with pLVX-vector or pLVX-LINC01050, as determined by CCK-8 assays. Data are presented as mean ± SD (n = 3). **P* < 0.05.


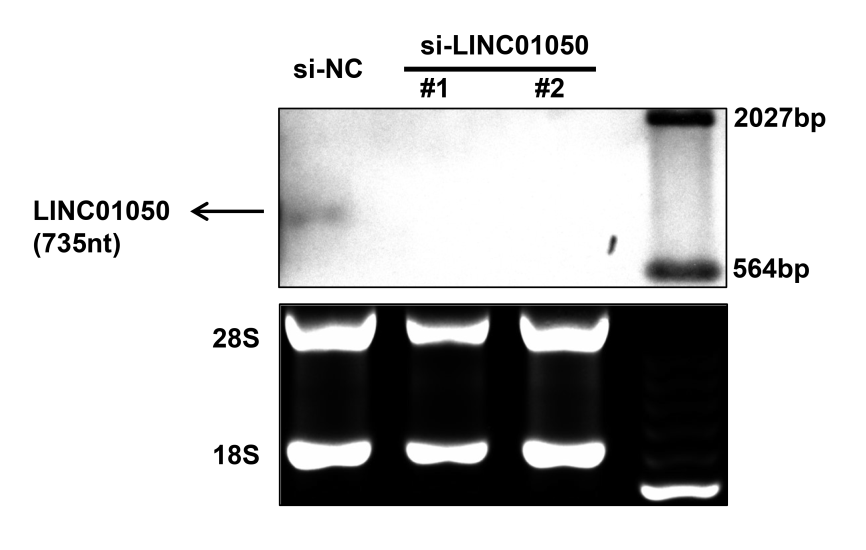


**Figure S5** Northern blot analysis of LINC01050 in gastric cancer KATO III cells with RNA probe. KATO III cells were transfected with si-NC, si-LINC01050#1, or si-LINC01050#2 for 24 h.

**
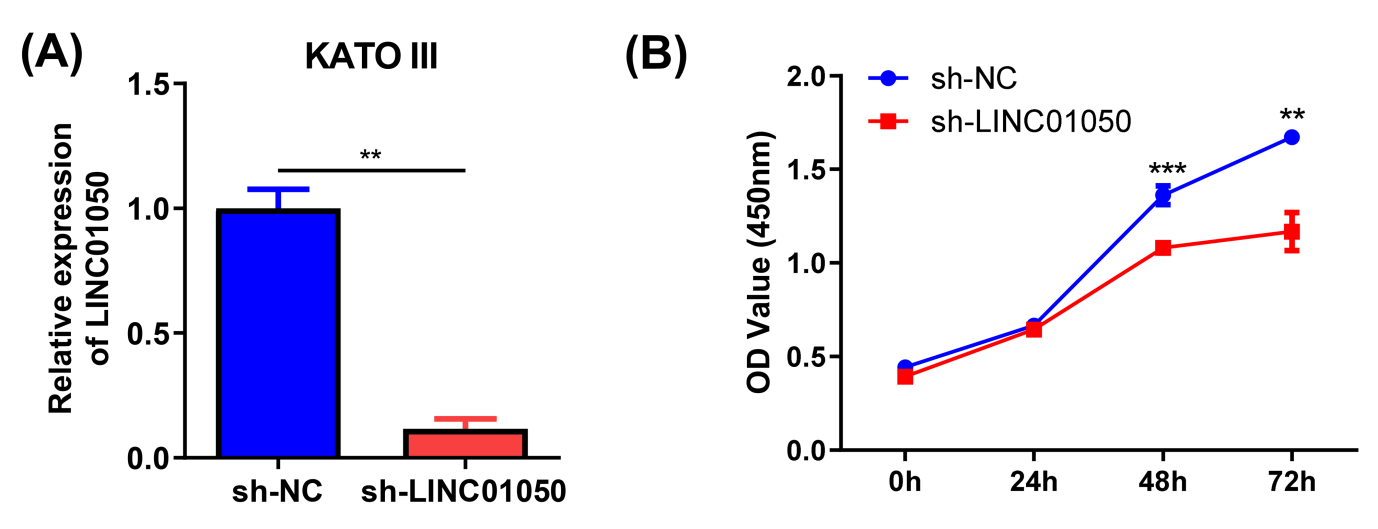
**

**Figure S6** Knockdown of LINC01050 by shRNA inhibits gastric cancer cell proliferation. **a** qRT-PCR analysis of LINC01050 expression in BGC823 cells transduced with lentiviral sh-NC (negative control) or sh-LINC01050. Data are presented as mean ± SD (n = 3). ***P* < 0.01. **b** The proliferation of BGC823 cells transduced with lentiviral sh-NC or sh-LINC01050 was determined using CCK8 assays Data are presented as mean ± SD (n = 3). ***P* < 0.01, ****P* < 0.001.


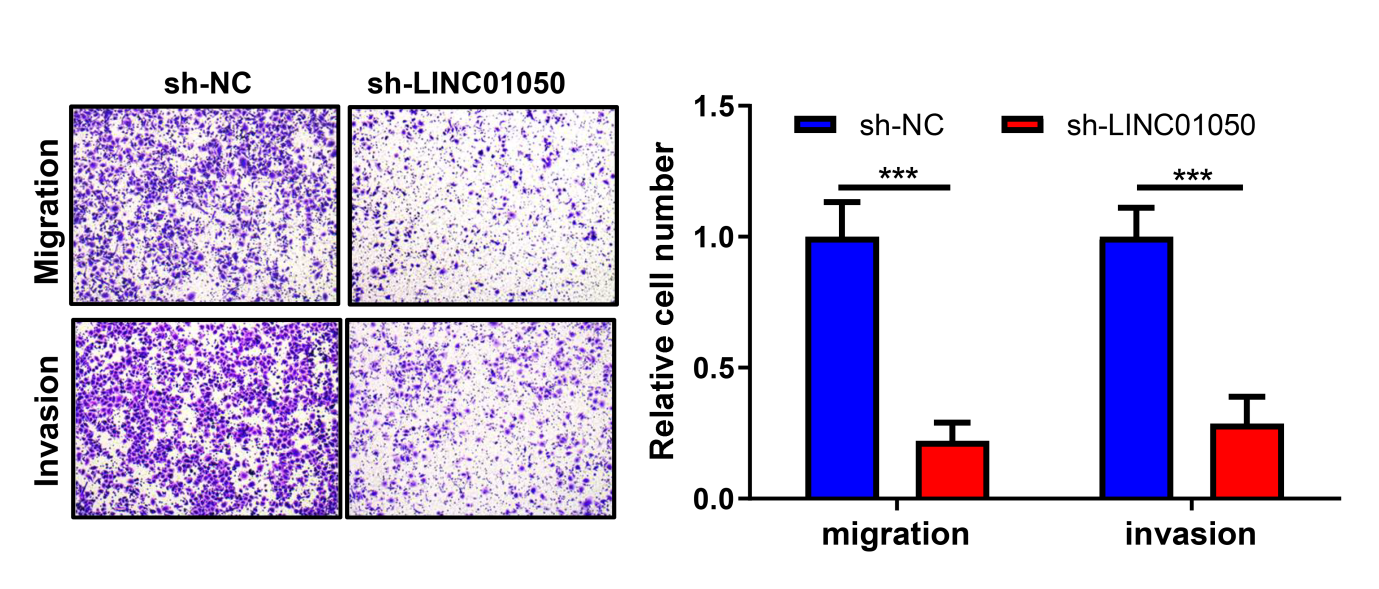


**Figure S7** The migration and invasion capabilities of BGC823 cells transduced with lentiviral sh-NC or sh-LINC01050 were assessed by Transwell assays. The data are presented as the mean ± SD. ^***^*P* < 0.001.


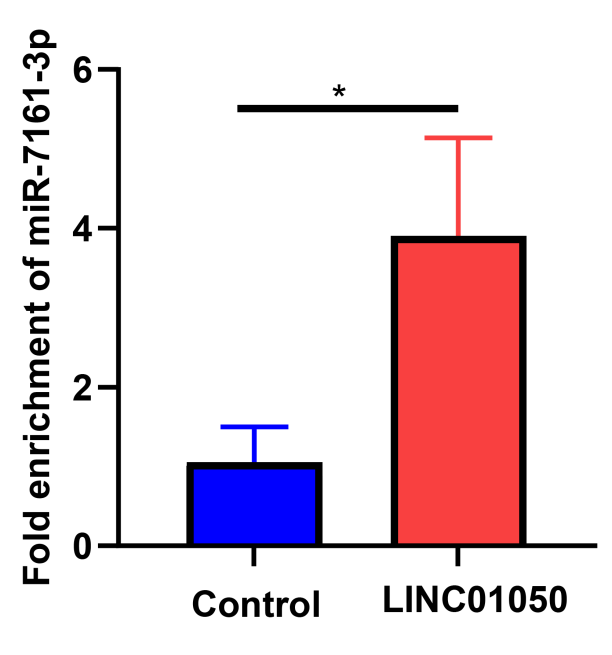


**Figure S8** RNA pull-down assay was performed in KATO III cells. miR-7161-3p level pulled down by the LINC01050 biotin-labeled probe was assayed by qRT-PCR. **P* < 0.05.


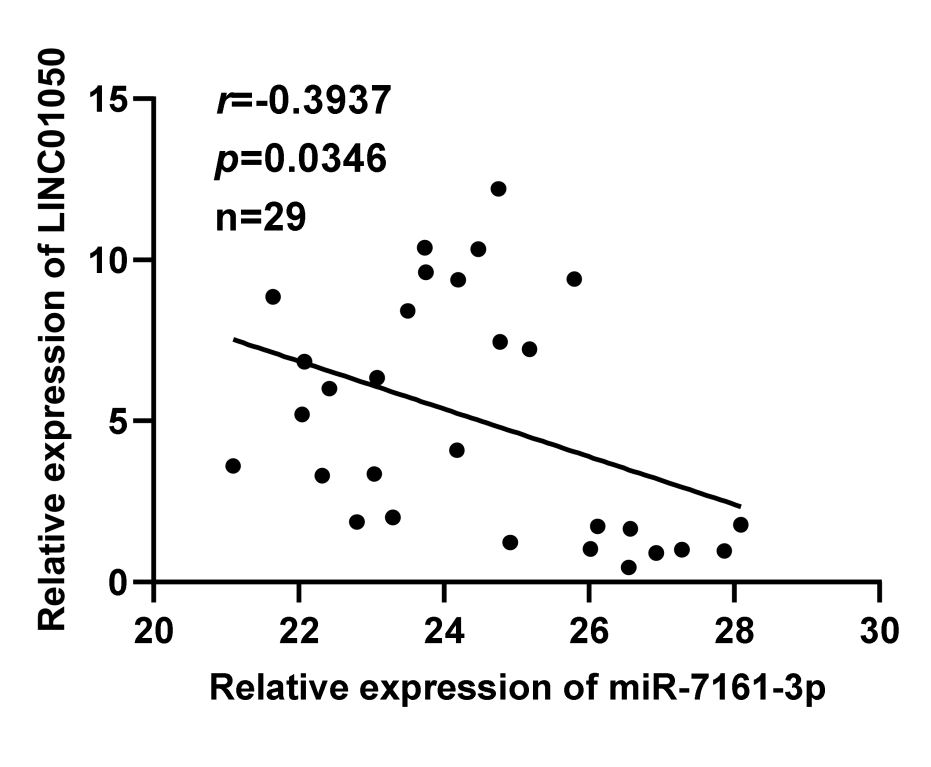


**Figure S9** The relationship between LINC01050 and miR-7161-3p expressions in gastric cancer tissues. The expressions of both LINC01050 and miR-7161-3p were detected in gastric cancer tissues obtained from 29 clinical patients by qRT-PCR.


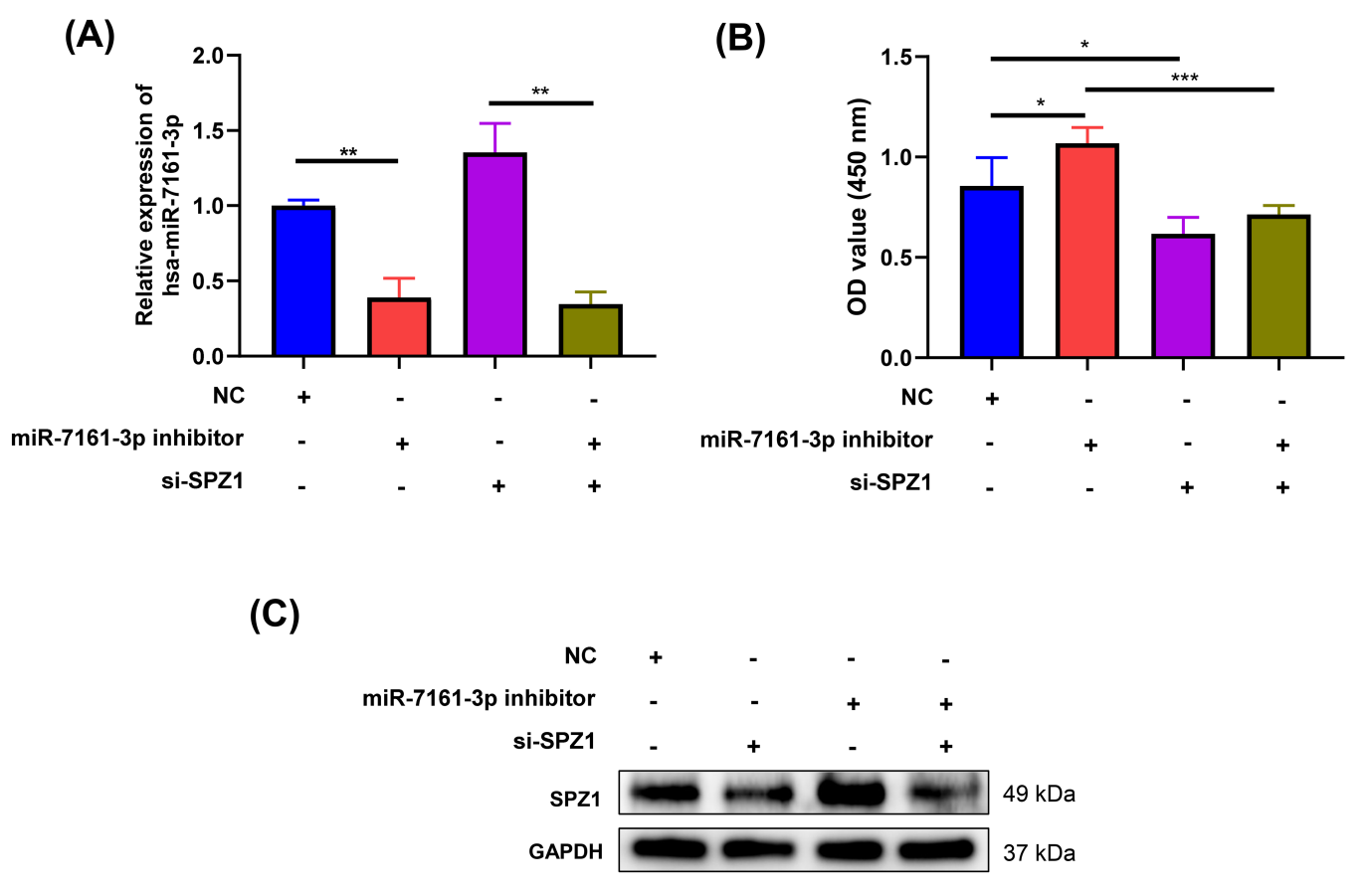


**Figure S10** Growth of KATO III cells transfected with the negative control (NC), miR-7161-3p inhibitor, si-SPZ1 or miR-7161-3p inhibitor plus si-SPZ1. **a** The expression of miR-7161-3P was determined by qRT-PCR. **b** Cell growth was determined by CCK8 assays. **c** The expression of SPZ1 protein was detected by Western blotting. Data are presented as mean ± SD (n = 3). **P* < 0.05, ***P* < 0.01, ****P* < 0.001.


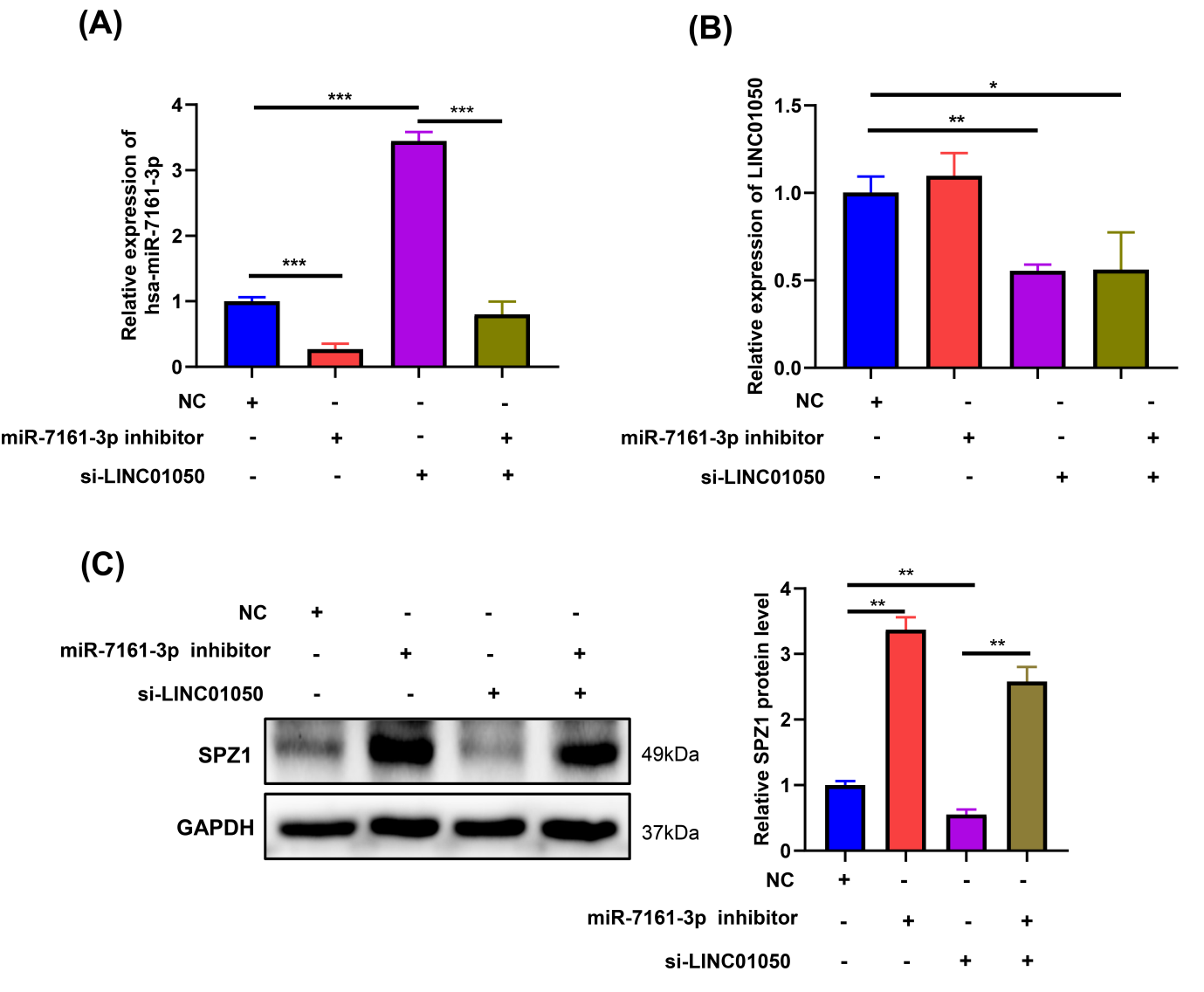


**Figure S11** miR-7161-3p inhibitor treatment reverses the LINC01050 knockdown-induced growth inhibition. **a-b** The expression of miR-7161-3p and LINC01050 was determined by qPCR. **c** Western blot analysis of SPZ1 protein level in KATO III cells transfected with NC, si-LINC01050, miR-7161-3p inhibitor or si-LINC01050 plus miR-7161-3p inhibitor. Data are presented as mean ± SD (n = 3). ***P* < 0.01.

**
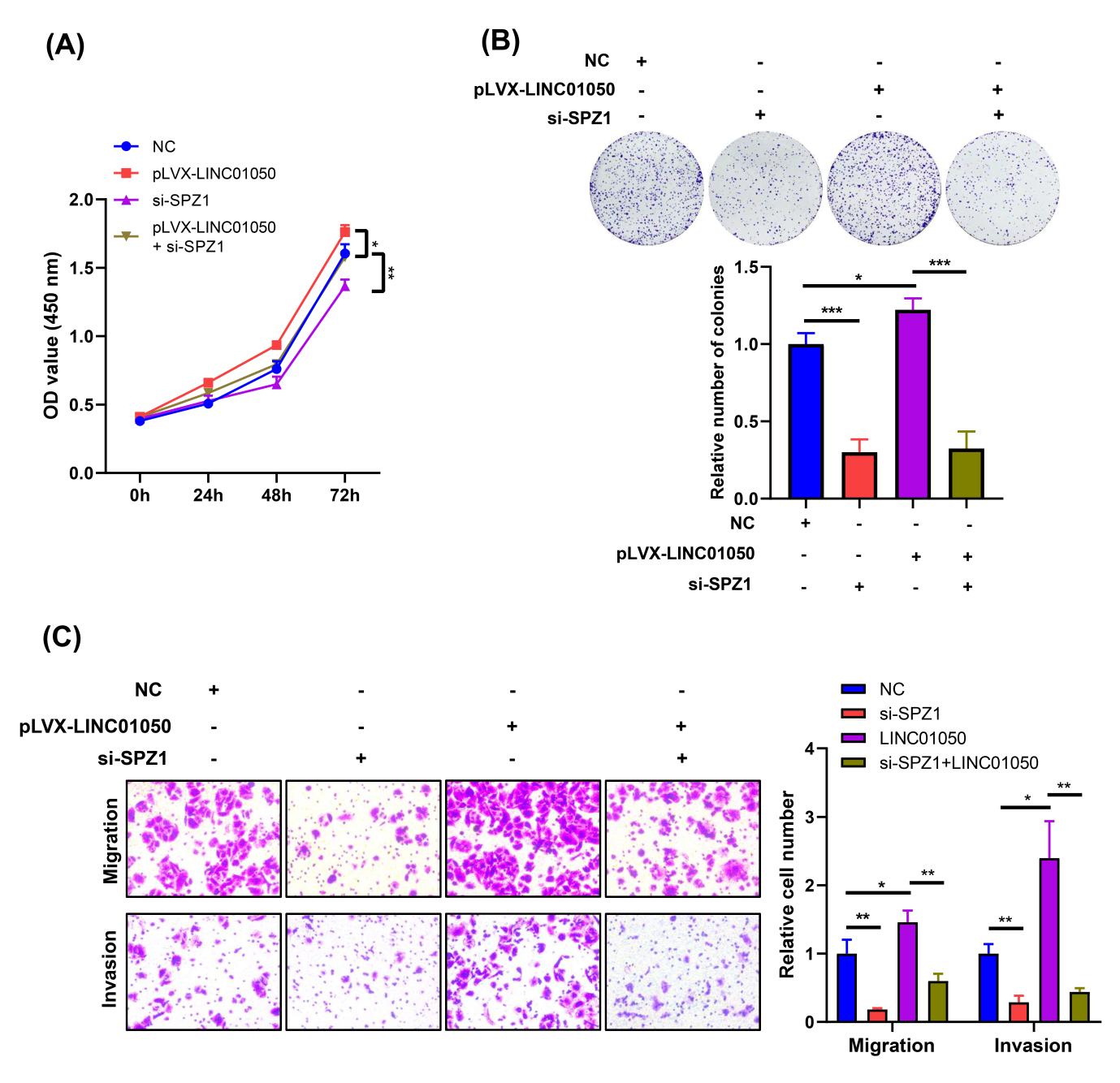
**

**Figure S12** Knockdown of SPZ1 reverses the phenotype induced by LINC01050 overexpression. **a** Growth curves of KATO III cells transfected with NC, si-SPZ1, pLVX-LINC01050 or pLVX-LINC01050 plus si-SPZ1 were revealed using CCK8 assays. **P* < 0.05, ***P* < 0.01. **b** The colony formation capabilities of KATO III cells transfected with NC, si-SPZ1, pLVX-LINC01050 or pLVX-LINC01050 plus si-SPZ1 were revealed. **P* < 0.05, ****P* < 0.001. **c** Migration and invasion capabilities of KATO III cells transfected with NC, si-SPZ1, pLVX-LINC01050 or pLVX-LINC01050 plus si-SPZ1 were revealed using Transwell assays. **P* < 0.05, ***P* < 0.01.

Figure S13 Association analysis of LINC01050 and c-Myc expression in TCGA data.
